# Material characterization of stone surfaces in the inner chambers of the Khufu (Cheops) Pyramid: towards informed conservation strategies

**DOI:** 10.1038/s41598-026-48805-8

**Published:** 2026-04-16

**Authors:** Clarimma Sessa, Randa Deraz, Olga Popovych, Mehdi Tayoubi, Mohamed Elkarmoty, Hany Helal, Christian U. Grosse

**Affiliations:** 1 Chair of Conservation-Restoration, Art Technology and Conservation Science, Technical University Munich, Oettingenstrasse 15, 80538 Munich, Germany; 2 UNESCO Chair on Science and Technology for Cultural Heritage, Faculty of Engineering, Cairo University, Gamaa Street 1, Giza, 12613 Egypt; 3 Chair of Non-destructive Testing, TUM School of Engineering and Design, Technical University of Munich, Lichtenbergstraße 2, 85748 Garching b. München, Germany; 4 Dassault Systèmes, 10 Rue Marcel Dassault, 78140 Vélizy-Villacoublay, France; 5 Heritage Innovation Preservation Institute (HIP Institute), 50 Rue de Rome, 75008 Paris, Île-de-France France; 6 Department of Mining, Petroleum, and Metallurgical Engineering, Faculty of Engineering, Cairo University, Gamaa Street 1, Giza, 12613 Egypt

**Keywords:** Khufu-Cheops Pyramid, Queen’s Chamber, King’s Chamber, Aswan granite, Soluble salts, Macro-X-ray fluorescence, XRD, SEM-EDX, Environmental sciences, Materials science, Solid Earth sciences

## Abstract

This study presents a systematic material characterization of the Queen’s and King’s Chambers stone surfaces of the Khufu Pyramid, conducted within the framework of the ScanPyramids mission. The two chambers show distinct interactions with their environment due to their differing lithologies. In the Queen’s Chamber, halite was identified as the main phase responsible for efflorescence on the massive limestone blocks. In the King’s Chamber, kaolinization of granite minerals appears to drive salt efflorescence formation in the upper blocks. Importantly, this research reports the first in situ application of X-ray fluorescence imaging for the characterization of Aswan granite. By mapping elemental distributions and correlating them with mineralogical phases, a completely non-destructive analysis of the granite surfaces was possible. In addition, a chemometric approach is proposed as an alternative to imaging for mineral-phase differentiation based on spot measurements. The study demonstrates how in situ portable non-destructive (contact-free) methods, combined with targeted micro-sampling, provide critical insights into deterioration mechanisms and support preventive conservation strategies for one of the world’s most iconic monuments.

**Supplementary Information:**

The online version contains supplementary material available at 10.1038/s41598-026-48805-8.

## Introduction

**Fig. 1:**
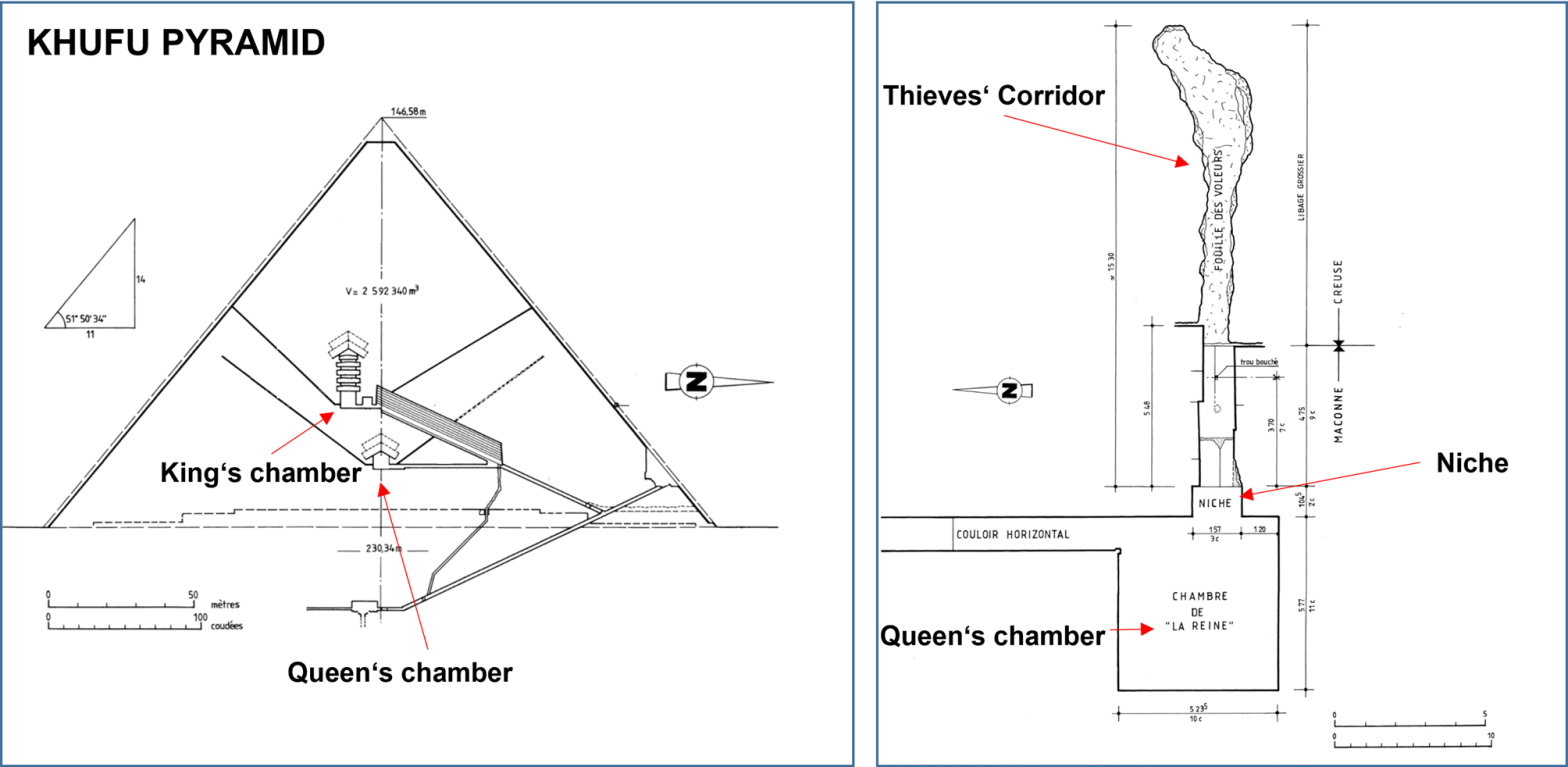
Schematic crosssection of the Khufu Pyramid (left); Queen’s chamber and Thieves’ corridor orthogonal projection (right, modified after Dormion^3^.

The Pyramids of Giza were built during the 4th Dynasty (c. 2575–c. 2465 BC) on a rocky plateau on the west bank of the Nile River near Al-Jīzah (Giza) in northern Egypt. The pyramids—Khufu (Greek: Cheops), Khafre (Greek: Chephren), and Menkaure (Greek: Mycerinus)—are named after the kings for whom they were constructed. The northernmost and oldest of the three was built for Khufu (2589−2566 BC), the second king of the 4th Dynasty^1^. Known as the Great Pyramid, it is the largest of the three pyramids, with each side of its base originally measuring an average of 230 m and its original height reaching 147 m^2^. Figure 1 shows its schematic cross-section modified after Dormion^3^. Despite its immense size, the pyramid contains relatively few inner open spaces. A sloping corridor descends from the entrance through the pyramid’s internal masonry. Branching off from this descending corridor is an ascending passageway that leads to the Queen’s chamber, as well as a large slanting passage known as the Grand gallery. Little-known corridors, the so-called Thieves’ corridor, begin at the niche in the Queen’s chamber (Fig. 1b). At the upper end of the Grand gallery, a long, narrow passage provides access to the burial room, typically referred to as the King’s chamber. Further voids were detected in the latest years, thanks to the intensive scientific studies within the ScanPyramids mission (SPM, www.scanpyramids.org)^4–6^.

Non-destructive testing methods—including ground-penetrating radar, ultrasonic testing, and electrical resistance tomography—have been employed to further investigate their internal structure, with the aim of localizing and characterizing the geometry of hidden chambers and corridors^6–8^. Investigating the structure of these still enigmatic monuments enabled direct access to their material fabric.

In 2022, a multidisciplinary team of heritage scientists and conservators from Cairo University (CU) and the Technical University of Munich (TUM) initiated, in frame of the SPM, in collaboration with the responsible of the Pyramids Antiquities Area and under authorization of the Permanent Scientific Committee of the Supreme Council of Antiquities, a systematic assessment of the conservation state of the inner spaces of the Khufu Pyramid, with particular focus on the Queen’s and King’s Chambers. Detailed observations revealed the presence of efflorescence phenomena on some areas of the wall surfaces of both chambers, as well as localized black deposits formation within the Thieves’ corridor.

This paper presents the documentation and conservation assessment of the above-mentioned inner areas and aims to demonstrate how in situ, non-destructive analytical methods can be effectively employed to monitor soluble-salt distributions on historical stone surfaces, thereby enabling more selective and representative sampling strategies. An in-situ measurement campaign was carried out to investigate the stone composition using X-ray fluorescence spectroscopy (XRF), complemented by targeted and strictly limited sampling of salt formations for laboratory-based analyses. National heritage laws, particularly in Egypt, impose strict regulations on the investigation, conservation, and sampling of archaeological sites, making non-invasive approaches indispensable for on-site material assessment^9–11^. In this context, XRF enabled a first systematic chemical characterization of the stone material across relatively extended surface areas, as well as of the associated salt components, thereby providing essential baseline data to support and justify a subsequent sampling campaign. The analyses of all the samples, in accordance with local laws, were conducted exclusively in Cairo at the Laboratory of Raw Building Materials and Processing Technology Research Institute, employing Scanning Electron Microscopy (SEM) coupled with Energy-Dispersive X-ray Analysis (EDX) and X-ray Diffraction (XRD).

The conservation of Egyptian archaeological sites faces many challenges, particularly regarding salt efflorescence and other forms of degradation of natural stone^12–15^. The Pyramids of Giza, with their monumental limestone and granite architecture, have long conveyed an impression of exceptional durability. However, increasing anthropogenic pressure associated with high visitor numbers, combined with evolving microclimatic conditions driven by climate change, is progressively endangering their integrity^12,16,17^.

Already in 1997, Slayman wrote in his short contribution “The Pyramid Closes”: *The chambers inside the Pyramid of the pharaoh Menkaure (2532 − 2504 B.C.) at Giza have been closed to visitors for cleaning and repair. Moisture exhaled by tourists had raised the humidity within them to such high levels that salt from the stone was crystallizing*,* and fungus was growing on the walls. Left unchecked*,* the salt could cause the stone to crack*,* weakening the structure. A ventilation system may be installed to help keep humidity as low as possible. The pyramids of Cheops and Khafre are already closed for similar work*^18,19^. At that time, the inner areas of the pyramids were cleaned, and new electrical and ventilation systems were installed, a necessity also highlighted by the Egyptologist Zahi Hawass in an interview with *The New York Times*^20^. Gandah (2019) discusses the critical condition of the Pyramid of Khufu and cites the work of Jean Kerisel, who, as early as 1981, investigated the impact of approximately 6000 daily visitors entering the pyramid. Based on Gandah, Kerisel documented the resulting strong entropic degradation, describing the enrichment of water vapor, carbon dioxide, and heat, which—depending on seasonal conditions and temperature—enhance condensation on the interior walls. In contrast, the Giza Plateau now receives approximately 38,000 visitors per day, indicating a marked increase in anthropogenic pressure compared to the conditions reported by Kerisel, although precise data on access to the pyramids’ interiors are unavailable.

If the humidity in such sites is not solely due to visitors, then their presence surely influences its variability and fluctuations^21,22^.

Soluble salts are among the most important causes of stone degradation. Salts damage stone in several ways, the most critical being the growth of salt crystals within the pores, which can generate sufficient stress to overcome the stone’s tensile strength and turn it into powder. The model of the disruption process by crystallizing salts has been extensively described, for example, by Zehnder and Arnold^23^. As the saline solution evaporates, aggregates and granular crusts form in the larger pores. The cohesion or strength of the host material is then reduced by disruption along cracks caused by the crystallization pressure of clustered aggregates growing on pore walls. Salt crystallization is controlled by relative humidity (RH) and temperature. As RH decreases, water evaporates from a salt solution until the deliquescence relative humidity (DRH) of the specific salt is reached, at which point the solution becomes saturated; below the DRH, the salt crystallizes. Conversely, when RH exceeds DRH, the salt absorbs water vapor from the air and dissolves, forming a saturated solution that becomes progressively diluted as RH continues to rise. For materials like stone, especially for very porous ones, repeated cycles of deliquescence and recrystallization (caused by RH fluctuations around the DRH) can cause significant physical stress, leading to material degradation^22,24^. According to Hemeda et al., the limestone blocks of the three Giza pyramids exhibit a relatively high total porosity, ranging from 21% to 33%, which hs important implications for moisture transport and salt-related deterioration processes^12^.

It is important to stress that humid conditions not only promote the mobilization of soluble salts but also favor chemical reactions between the stone and airborne pollutants. The presence of primary (e.g., SO_2_, NO_x_, CO_2_) and secondary (e.g. ozone, nitrates) pollutants inside may affect stone through sulfation, deposition, and particle accumulation and/or the creation of favourable conditions for microorganisms, which can form biofilms on the surfaces of stone^25,26^. Specifically, on calcareous stones, air pollution and dust may lead to the formation of black crusts in sheltered zones. Black crusts result mostly from a sulfation reaction (SO_2_ reacts with calcite to form gypsum, CaSO_4_·2H_2_O) and from the entrapment of particles (especially soot, dust), which causes their blackening. The black appearance of calcareous stone may also result from soiling and/or incrustation^27^. Furthermore, indoor human sources of ammonia are correlated to emissions from breath, skin, sweat, etc.^28^. All the factors addressed above can have a synergistic effect, accelerating the degradation of natural stone.

An essential step in understanding the possible causes of natural stone degradation, particularly when salt-related decay is involved, is the characterization of both the stone composition and the nature of the salts present. In this study, in situ XRF analysis was employed to document the presence of salts in areas where crystallization was not yet macroscopically evident. The use of non-destructive analytical methods offers the significant advantage of enabling large numbers of measurements, thereby enabling the investigation and monitoring of relatively extensive surface areas. When applied with a macro-scanning setup using an X–Y stage—as was possible in the King’s Chamber—XRF enables the mapping of different mineral phases and the assessment of material heterogeneity, particularly in complex lithologies such as granite^29^. In situations where scanning is not feasible, a large number of discrete measurements can still be acquired; however, data interpretation becomes increasingly complex. In such cases, chemometric approaches provide an effective tool for interpreting multivariate datasets and tracing the compositional variability of different mineral phases^30^. Consequently, this paper proposes a straightforward, easily applicable chemometric approach for the management and interpretation of XRF data for granite analysis.

Despite early observations in the literature, to the best of our knowledge, no scientific publications providing analytical data on the nature of the salts and/or the mechanisms of their formation in the Khufu pyramid’s interior spaces are currently available. Therefore, the present study marks a significant step forward in understanding the degradation pathways and underlying causes affecting the internal surfaces of the chambers, which is essential for planning informed preventive conservation strategies for one of history’s most iconic monuments.

## Materials and methods

### Sampling

A total of nine samples were collected from the Queen’s Chamber and the Thieves’ Corridor behind the niche. Sample descriptions and documentation are provided in Supplementary Table 1. Sampling locations were selected based on the presence of visible salt efflorescence and/or black deposits, ensuring a minimally invasive approach. In addition, naturally detached materials with well-documented provenance were analyzed. The samples were analyzed by means of laboratory analytical techniques described below. Efflorescence observed on the upper blocks of the King’s Chamber was not sampled due to limited accessibility and the absence of sampling permission for this area.

### Analytical methods

#### X‑ray fluorescence (XRF)

The XRF measurements were performed using a portable X-ray fluorescence spectrometer (ELIO, Bruker) with a Rh target (10–50 kV excitation), and a 17 mm^2^ SDD detector. The focal spot size is 1 mm^2^. The tube voltage and anode current were set to 40 kV and 90 µA. Calibration measurements were carried out using an AgCu reference at the start of each measurement day. No helium flow or filters were used. Thanks to an x/y scanner, the instrument allows mapping up to a maximum area of 10 × 10 cm. Data were processed using Bruker Elio software, release 1.6.0.64. Measurement parameters were optimized to balance acquisition time and spatial coverage, while accounting for the overall campaign duration and the restricted access time within the chambers. Spot locations were systematically recorded and documented, enabling future repeat measurements to assess temporal changes and the potential development of salt efflorescence. Detailed data of the measurement locations are available in the supplementary material (Supplementary Fig. S3).

**Fig. 2:**
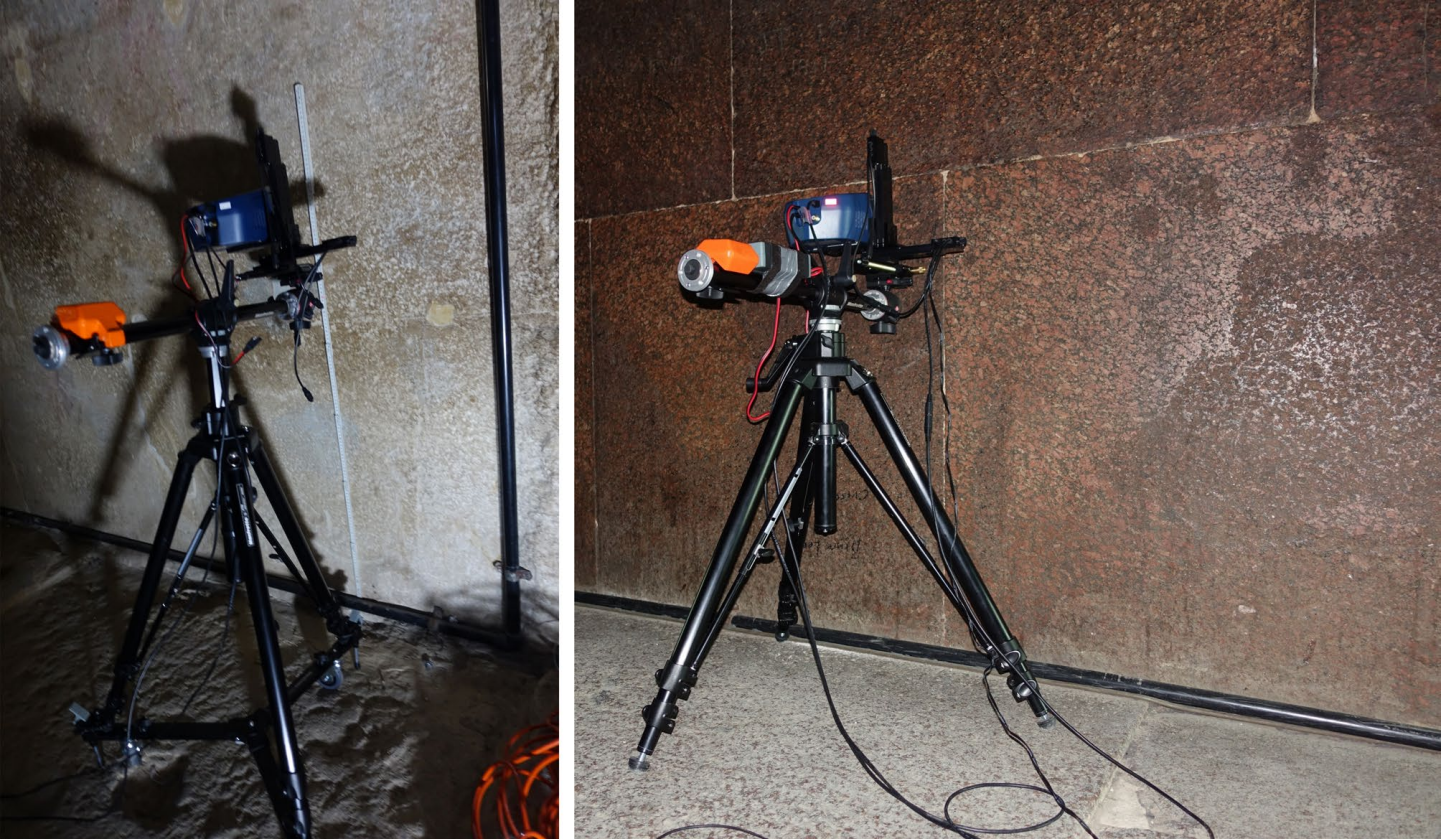
XRF spectrometer during the measurement in the Queen’s chamber (left) and King’s chamber (right).

In the Queen’s Chamber, a minimum of two measurement spots (tot. 32 spots) were selected per block, deliberately avoiding areas affected by anthropogenic alteration (e.g., graffiti). Due to logistical constraints, measurements could be conducted only on the first and second courses of blocks from the floor of the western and northern walls (Fig. 2, left). A long acquisition time of 180 s per spot was adopted to improve counting statistics and signal-to-noise ratios for low-Z elements.

The scanning function was employed exclusively in the King’s Chamber, where a secondary power supply was available; in all other locations, the instrument operated on external battery power. Seven maps (10 × 10 mm, 3 s acquisition time per pixel) were acquired on the southern wall of the King’s Chamber. In addition, 39 discrete spot measurements were acquired with a longer acquisition time (180 s) to improve signal-to-noise ratios and elemental sensitivity. Due to logistical constraints and the presence of visitors, measurements could be conducted only on the first courses of blocks of the western, northern, and southern walls (Fig. 2, right). Micro-photographs acquired by the camera of the instrument, together with detailed on-site documentation of each measurement location, provided a precise phenomenological context (e.g. colour of the mineral phase) for the analyzed surfaces. Semi-quantitative data evaluation was performed with ArtTAX-Ctrl software (Intax). PCA was applied using a PLS_Toolbox (Eigenvecton Research) running on MATLAB R2022a. The data were mean-centered and pretreated by applying a standard normal variate (SNV) algorithm (Burns and Ciurczak 2007).

#### X-ray diffraction (XRD)

The analysis was commissioned to the Raw Building Materials and Processing Technology Research Institute, Housing and Building National Research Center (87 El-Tahrir St., Dokki, Giza 11511, P.O. Box: 1770 Cairo, Egypt). The qualitative mineralogical composition of selected samples was determined by a X’Pert PROPW3040/60 PANalytical model diffractometer equipped with a monochromatic Cu–Kα radiation source at a wavelength of 1.54060 Å. The analysis was performed at operating conditions of 40 kV and 30 mA over a scanning range of 0–60° (2θ). The 2θ accuracy of the diffractometer is ± 0.03°, and the detection limit is below 5 wt% for any mineral phase. Identification of the contained mineral phases was performed using the X’Pert HighScore software 2006 and the PDF-2 database.

#### Scanning electron microscopy (SEM) with energy dispersive X-ray analysis (EDX)

The analysis was commissioned to the Raw Building Materials and Processing Technology Research Institute, Housing and Building National Research Center (Dokki, Giza, Cairo, Egypt). The FEI Quanta 250 FEG-SEM is equipped with a Scotty field emission gun and an Everhart-Thornley detector for secondary electrons to deliver ultrahigh resolution (1.2 nm @ 30 kV), a backscattered electron detector in high vacuum mode, and a large field secondary electron detector for low vacuum operation (3.0 nm@ 30 kV) imaging. The Quanta 250 integrates with EDAX detectors. Accelerating voltage 200 V–30 kV; Operating Voltage 5–30 kV operating voltage; Magnification: 30X–300 kX. SEM–EDX analyses were performed using standardless quantification with ZAF matrix corrections. To minimize charging effects, the samples were coated with a thin graphite layer prior to analysis. Each sample was then subdivided into three analytical zones—surface, internal, and inner core—to assess potential compositional variability within the material.

#### Data loggers and CO_2_ sensor

Environmental parameters such as Temperature (T) and Humidity (RH%) were monitored using data loggers Testo 174 H Set—Mini Data Logger with a measurement range between − 20 °C to + 70 °C (accuracy ± 0.5 °C, resolution 0.1 °C) as well as 0 to 100% RH (accuracy ± 3%RH − 2 bis + 98%RH − bei + 25 °C; resolution 0.1%). Furthermore, a multi-parameter sensor capable of recording carbon dioxide (CO_2_) concentration, relative humidity (RH), and air temperature was used. The measurement range for CO_2_ was 0–6000 ppm (resolution 1 ppm), 10–95% for RH (resolution 0.1%), and a range of − 20 to 60 °C for T (resolution 0.1 °C).

## Results and discussion

The conservation issues, as well as the analytical results obtained in the two chambers, will be discussed in two separate sections.

### Queen’s Chamber and thieves’ corridor

**Fig. 3:**
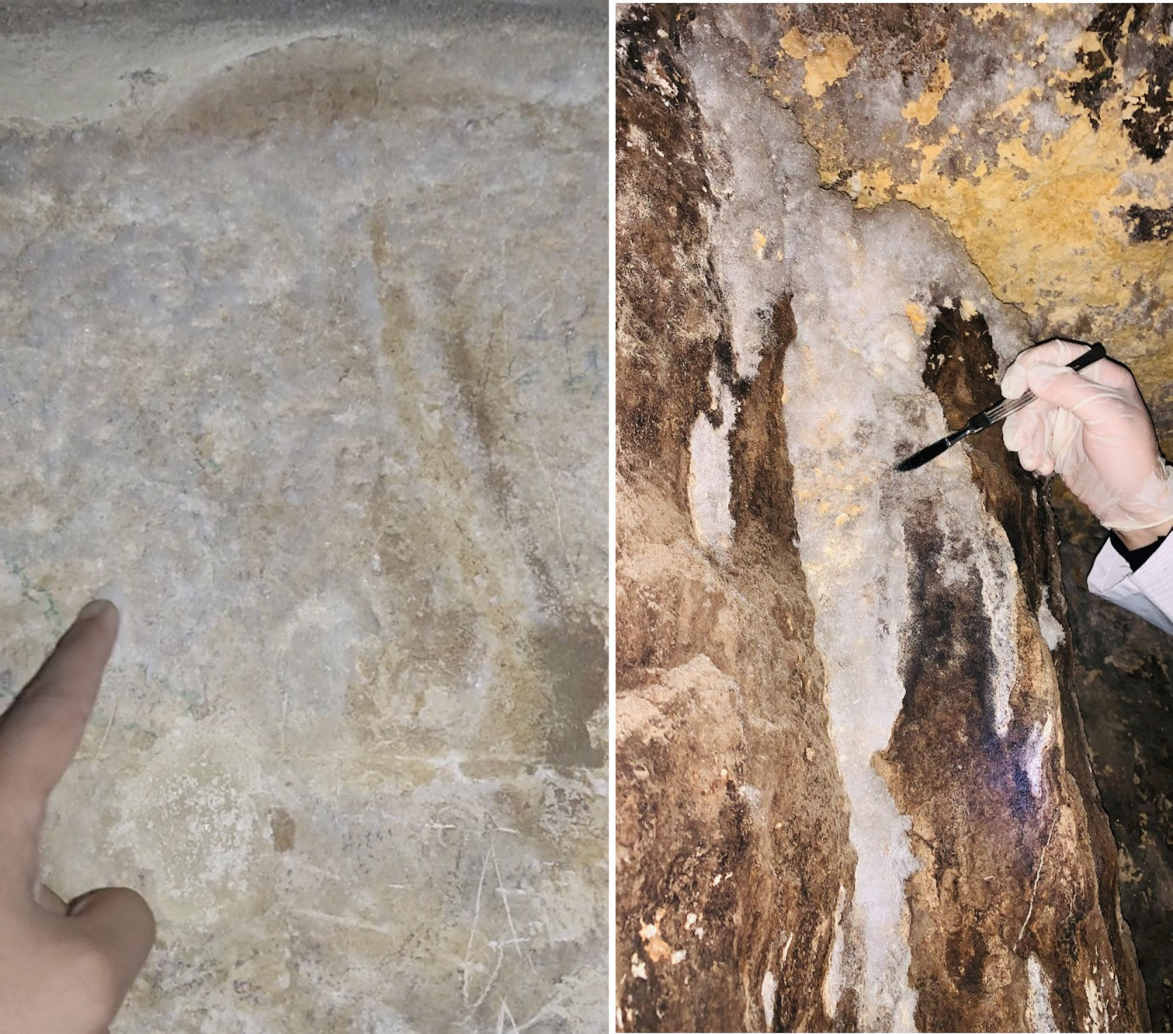
Salts formation on the western wall of the Queen’s Chamber in 2024 (left). In these areas, no salt efflorescence was visible during the XRF measurements campaign in 2022; Black areas and salts efflorescence at the end of the Thieves’ Corridor during the sampling in 2022 (right).

The Queen’s Chamber is centrally located within the Great Pyramid and is entirely built of limestone. It measures approximately 5.77 m in length, 5.23 m in width, and about 6.26 m in height^3^. Unfortunately, documentation of the conservation interventions carried out between 1995 and 1997 is not available^18,19^; however, based on published photographs and more recent documentation, it is evident that surface cleaning and the filling of wall cavities were carried out. In 2022, salt formation was observed only in very limited areas, serving as an early warning of incipient degradation processes. Since then, the situation has changed markedly, and extensive salt efflorescence is now visible on the stone surfaces. For example, the area shown in Fig. 3 (left, documented in 2024) did not exhibit efflorescence in 2022, when the surfaces of the northern and western walls were free of any salt formation. A similar trend is evident in Supplementary Fig. S4, which shows a marked increase in salt efflorescence on the ceiling between 2022 and 2024. In contrast, black deposits/areas were already present at the end of the Thieves’ Corridor in 2022 (Fig. 3, right). Anthropogenic alterations, including graffiti (residues of blue, green, and red colorants) and surface scratches, remain visible (Supplementary material Fig. S5). Notably, these features can also be identified in images published by Gilles Dormion in 2004^3^, indicating that they predate the 2004 documentation and may represent residues remaining after the cleaning interventions.

#### In-situ XRF analysis

**Fig. 4:**
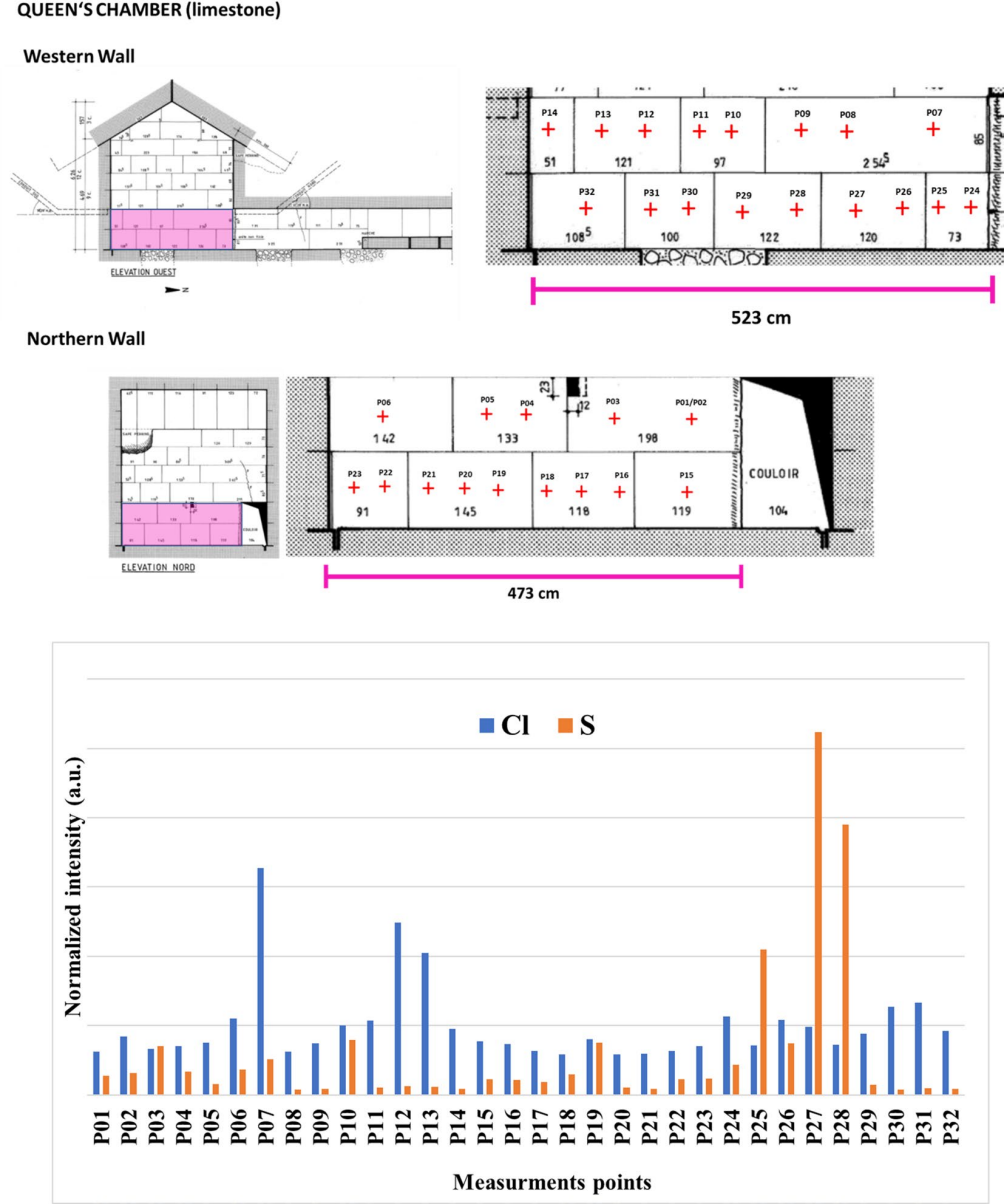
XRF measurement locations (upper). More information is available in Supplementary Fig. S3. Histogram of the total counts for selected characteristic lines of each element (Cl, S), normalized to the Rh Rayleigh peak (Kα_1_ emission line at 20.216 keV) (lower).

In 2022, systematic XRF analysis of the western and northern walls of the chamber was conducted in areas where no salt efflorescence was observed. The major and minor elements detected—calcium (Ca), iron (Fe), strontium (Sr), as well as titanium (Ti), silica (Si), sulphur (S), and chlorine (Cl)—are consistent with the previously published composition of limestone of the Khufu pyramid^31^. It is essential to note that light elements, such as Mg and Na, cannot be detected by XRF when operated in ambient air. Although a helium flush would have significantly improved sensitivity to these elements, it was not feasible given the logistical and safety constraints. The presence of S and Cl is of particular interest, as these elements may serve as indicators for potential formation of salts such as calcium sulfate, other sulfur-based phases (CaSO_4_) and other chlorine-based phases, most common being the sodium chloride (NaCl), both of which are commonly associated with efflorescence and sub- and surface degradation phenomena^15,32^. The XRF-detected signal intensities for S and Cl were high and varied across the surveyed blocks (Fig. 4), likely associated with the upward migration of soluble salts and their progressive accumulation at or near the surface^33^. It is important to consider that the XRF penetration depth depends on both the excitation energy of the detected element and the substrate density. As a result, the detection of light elements such as chlorine is highly surface-sensitive, with the measured signal primarily reflecting concentrations in the uppermost layers of the stone^34,35^. Notably, sulfur counts showed a marked increase only at XRF measurement points P27 and P28 (Fig. 4). These two locations correspond to an area where a restoration intervention is clearly visible. A filling of a wall cavity in this area can be inferred by examining the images published in Dormion (Annex XIV in^3^). The localized enrichment in sulfur strongly suggests that the material used to fill the lacuna in the block was S-rich, likely containing gypsum or other sulfur-based constituents that were not part of the original stone fabric.

#### Samples investigation: SEM-EDX and XRD analysis

SEM-EDX analysis of naturally detached material from visually unaffected areas of the stone substrate (samples S1, S2, S3, Supplementary Table S2) revealed the presence of elements including carbon (C), oxygen (O), sodium (Na), magnesium (Mg), aluminum (Al), silicon (Si), strontium (Sr), phosphorus (P), sulfur (S), chlorine (Cl), potassium (K), and calcium (Ca). These data indicate that elements commonly associated with soluble salts, such as Na and Cl are probably inherent components of the stone matrix. The results are compatible with the composition of the backing limestone blocks of the Khufu Pyramid published by Hemeda et al. Calcite (CaCO_3_) has been described as the main component, accompanied by minor amounts of iron oxides and quartz (SiO_2_), as well as traces of dolomite (CaMg(CO_3_)_2_), opaque minerals, and halite (NaCl)^12^. Klemm and Klemm describe halite as the primary salt present in the nummulitic limestones of the Gyza plateaux, while gypsum and potassium salts appear only in minor amounts. They observe that the presence of halite within the stratigraphic layers suggests that it is part of the original sedimentary environment, likely precipitated from evaporating marine or lagoonal waters during the rock formation process^31^.

**Fig. 5:**
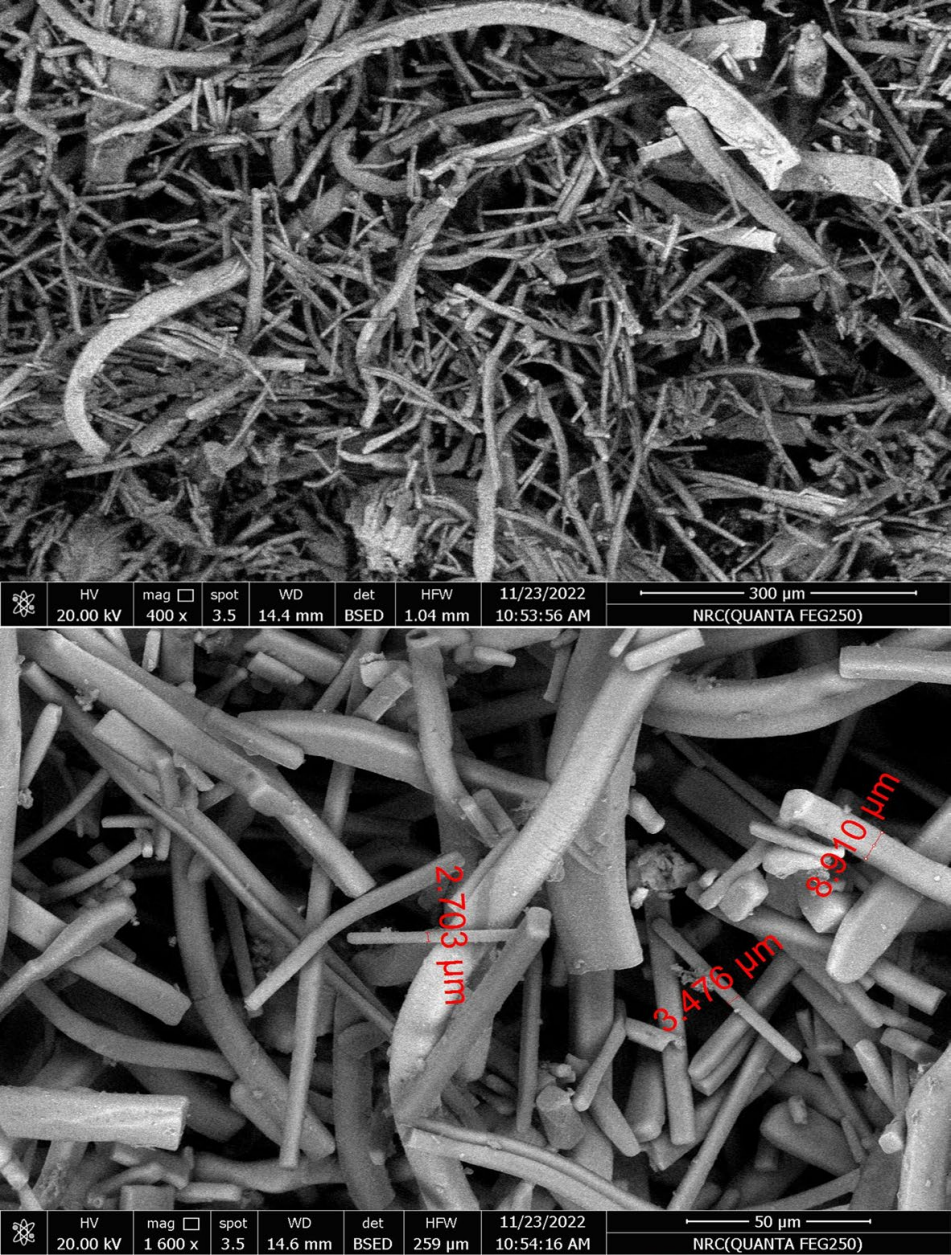
Typical halite whisker morphologies observed in SEM micrographs at 400× (upper) and 1600× (lower) magnification of sample S5.

Targeted analysis of the white efflorescence revealed a simpler elemental composition, with only Na and Cl detected, suggesting the predominance of a single soluble salt species (Samples S4, S5, Supplementary Table S2). Halite whiskers can be observed in the SEM images (400× and 1600× magnification, Fig. 5).

**Fig. 6:**
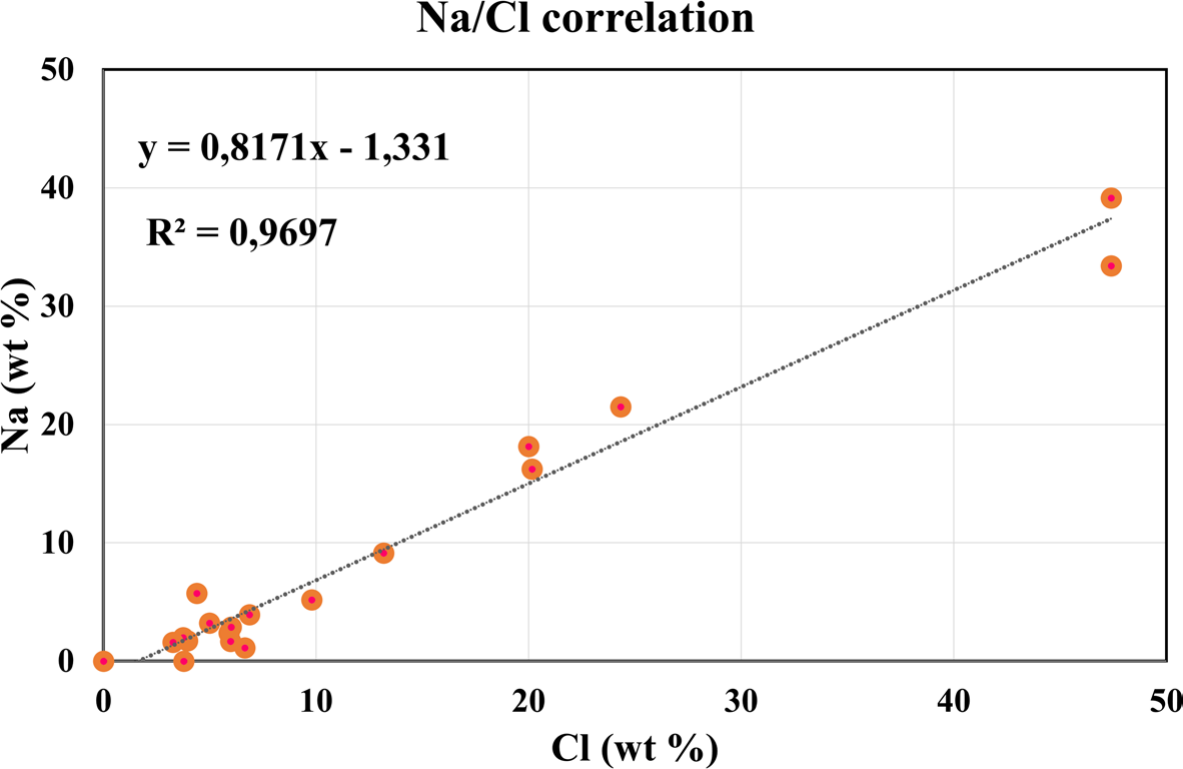
Na/Cl correlation regression graph based on SEM-EDX analysis.

A strong linear correlation (*R* = 0.969) was observed between the EDX-measured concentrations of Na and Cl across all samples, indicating the systematic presence of sodium chloride (Fig. 6).

**Fig. 7:**
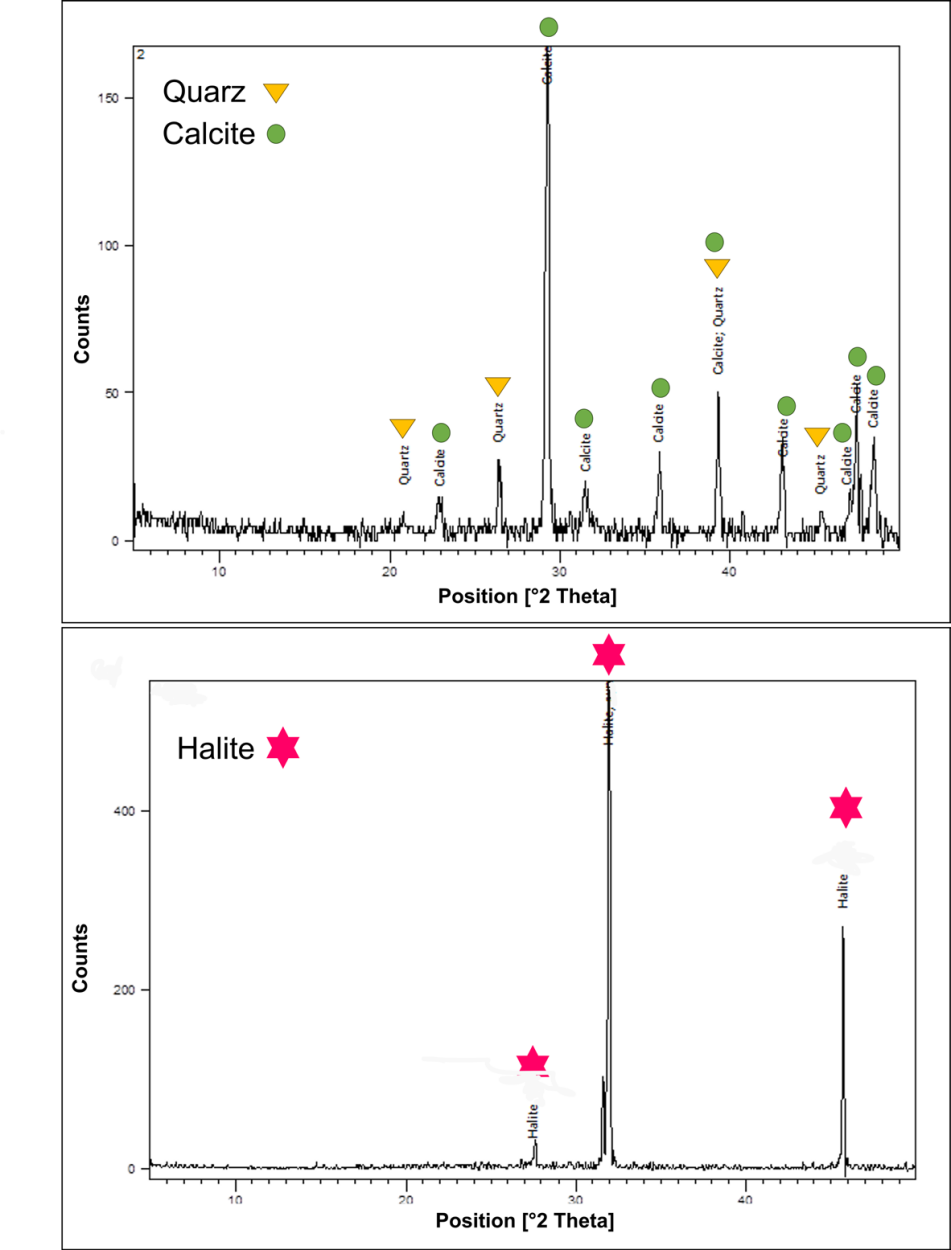
Exemplar XRD spectra obtained for samples S7 (upper) and S5 (lower).

The material obtained from the black areas at the end of the Thieves’ Corridor (sample S8; Supplementary Fig. S2) exhibited a chemical signature comparable to that of the naturally detached material not affected by salts (samples S1–S3). However, sulfur concentrations in sample S8 were markedly lower (≈0,6%) with respect to concentrations of approximately ≈ 3% in samples S1, S2, and S3. This observation suggests that extensive sulfation of the carbonate substrate, thus the formation of gypsum-bearing deposits, commonly defined as black crusts is unlikely. Further analysis could confirm this statement. The only sample exhibiting a higher sulfur signal (S7, S≈ 6%; Supplementary Table S2) corresponds to material recovered from the entrance of the Queen’s Chamber, which displays a brown coloration in contrast to the dark/black surface observed in the Thieves’ Corridor (Supplementary Fig. S2). Interestingly, sample S7 corresponds to a location where external pollutants may enter the chamber and/or where visitors are likely to exhale more forcefully after traversing the long, narrow corridor leading into the chamber and may be associated with gypsum-related degradation processes. In contrast, the XRD analysis of the same material detected only quartz and calcite (Fig. 7, upper). However, it should be considered that potential S-based phases may be below the detection limit. All the SEM-EDX analyses revealed a dominant presence of sodium and chlorine. These results indicate that, under current environmental conditions, there is no clear evidence of widespread gypsum formation and/or crystallization. These findings are corroborated by XRD analysis of the 7 samples, which confirmed the presence of only halite, calcite, and quartz (Fig. 7, down. Further XRD spectra are presented in Supplementary Fig. S7). No additional crystalline phases were identified in any of the analyzed specimens. Notably, no evidence of the presence of nitrate compounds was observed in any samples. In similar studies, Nazel^19^ reported nitrate salts in 20 samples collected from the first chamber of the Menkaure Pyramid; however, the article does not provide a detailed description of the sampling locations or the samples. Given that Slayman documented the presence of fungi in the Menkaure Pyramid, the occurrence of nitrates may also, in that case, be associated with biodegradation-related processes^36^. Based on our investigation, such phenomena have not been documented in the Queen’s Chamber of the Khufu Pyramid to date.

Considering the results presented above, it seems that the principal crystalline phase responsible for efflorescence is halite. In general, chlorides are considered highly detrimental to masonry and artifacts because they are highly soluble and hygroscopic^37^. They are the first salts to be dissolved when water condenses from the surrounding air. Furthermore, halite in solution is mobile and can penetrate, breaking up many crystalline structures during recrystallization. It is capable of retaining the adsorbed humidity in the masonry, and it lowers the transformation temperature of hydrate salts, facilitating the transformation from one hydration state to another, i.e., anhydrite to gypsum^38,39^.

The detection of sulfur does not allow for the unambiguous identification of specific sulfur-containing phases. It may indicate the presence of gypsum (CaSO_4_·2H_2_O), bassanite or hemihydrate (CaSO_4_·0.5H_2_O), anhydrite (CaSO_4_), or other sulfate-based salts such as sodium sulfate (Na_2_SO_4_). To confirm the presence and composition of these phases, further analysis would be necessary. The predominance of halite in surface crystals may be explained by its higher solubility relative to calcium sulfate phases, which may crystallize at different rates under specific environmental conditions^40,41^.

Halite has been frequently identified as a dominant salt contributing to efflorescence at numerous heritage sites across Egypt. For example, its presence has been documented at the Menkaure Pyramid^19^ and the Sphinx in Giza^42^. Similar occurrences were documented by Ahmed et al. at the White Monastery in Sohag^43^ and by Abdelaal et al. at the Imni Tomb in Lisht^44^. Comparable findings have also been reported at the Saqqara Pyramid^15^.

To prevent the crystallization of NaCl efflorescence on a limestone wall, the T and RH should be maintained at a stable level. Fluctuations in RH that cross the DRH threshold led to repeated cycles of salt dissolution and crystallization—processes that can cause significant physical damage to limestone substrates. Maintaining a stable temperature is also crucial, as temperature fluctuations directly affect RH levels. Notably, higher temperatures reduce the DRH of salts, meaning that even slight decreases in RH under warmer conditions can trigger salt crystallization, accelerating material deterioration. The critical parameters are guided by the DRH of NaCl, which is around 75%. Since the temperature measured in the chamber are between 20 and 40 °C, based on Steiger at al. the deliquescence humidities of sodium chloride should be between 75.4% RH (20 °C) and 75.2% RH (30/40 °C)^45^. It is important to stress that the critical relative humidity thresholds associated with NaCl crystallization can vary significantly depending on the specific case study and methodological approach. E.g., RH thresholds of 75.3% RH and 75.5%, respectively, as indicators for NaCl crystallization-dissolution cycles, have been considered by Grossi et al.^46^ and Sabbioni^47^. A relatively lower RH has been reported by Benavente et al. for a case study on the Postumius Tomb (Spain). They considered a critical relative humidity difference of 10% for NaCl, setting the RH threshold at 65.3%^48^. Thus, monitoring the surface conditions and microclimate within the Queen’s chamber will be a crucial next step for establishing optimal temperature and RH conditions. The fact that only halite has been detected could be a positive outcome of the study, as it simplifies establishing optimal environmental conditions. In fact, assessing the environmental risk of NaCl-related weathering can be quite challenging when complex salt mixtures should be considered^49^.

### King’s chamber

**Fig. 8:**
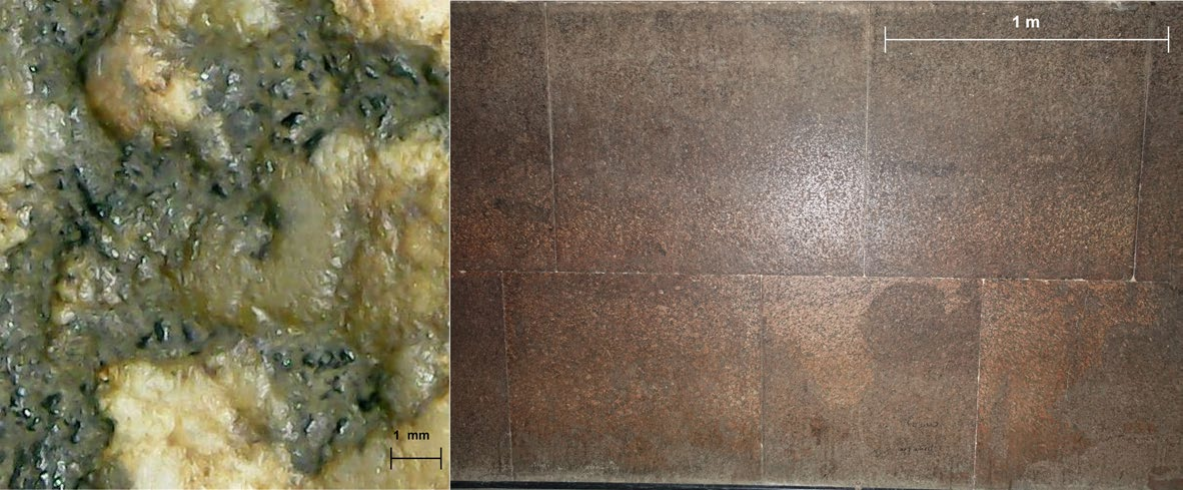
Microphotographs acquired with the XRF spectrometer cameras document greasy derivative deposits and surface discoloration (left), as well as traces of condensation droplets on the lower block courses, southern wall (right).

The King’s chamber is a rectangular room, measuring approximately 10.5 m in length, 5.2 m in width, and 5.8 m in height^3^. The chamber’s walls are composed of smooth, finely cut granite blocks. Based on macroscopic observation of mineral distribution and color the type of granite can be classified as red granite (Fig. 8, right). The rock’s grain allows recognition of the main crystalline phases based on their colour, simplifying identification of this material even without resorting to sampling^5^.

Some observations can be made regarding the condition of the surface and the chamber itself.

**Fig. 9:**
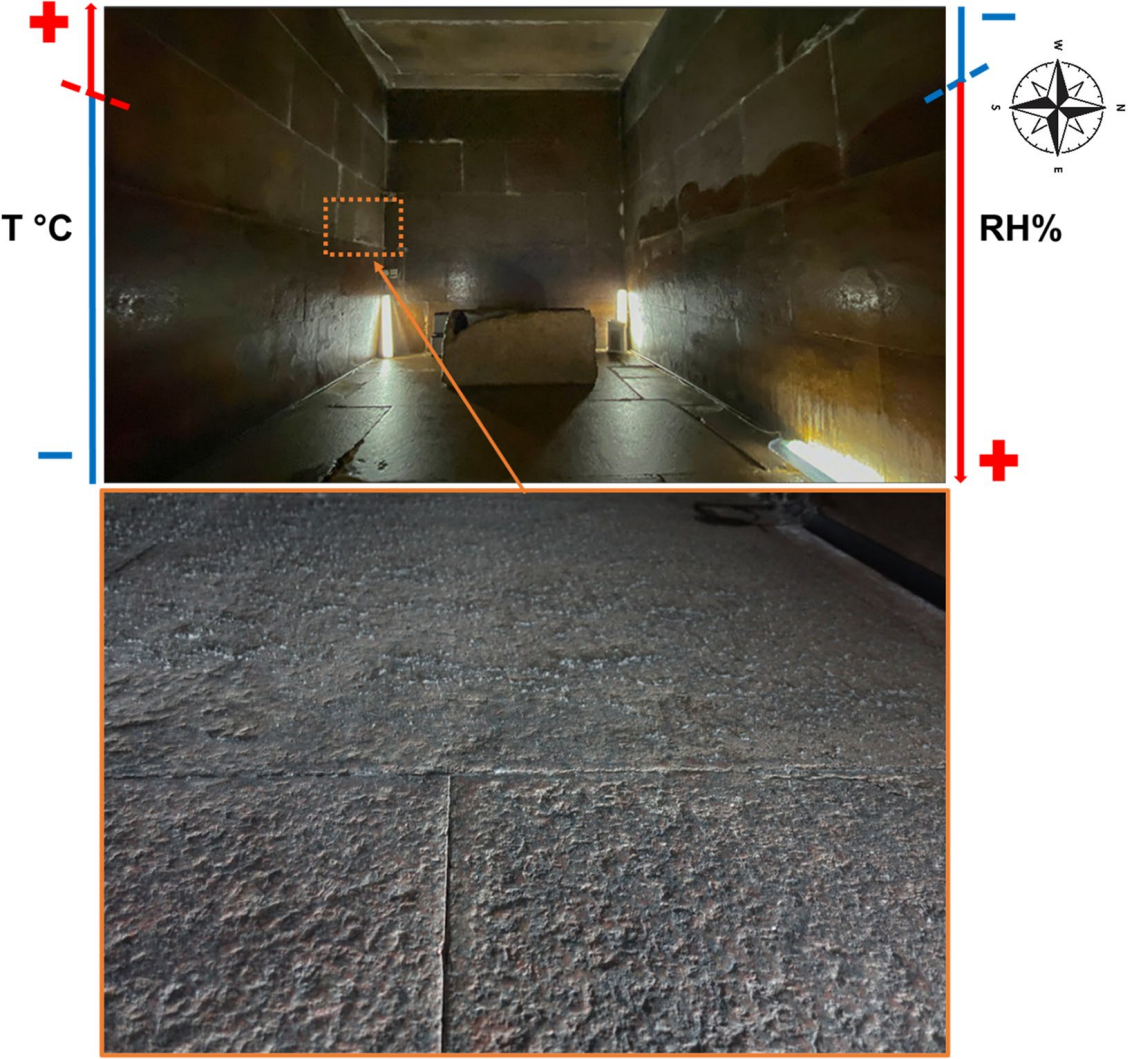
Overview of the western, northern, and southern walls of the King’s Chamber (upper, Photo of the King’s Chamber. Credits: Subhadip Mukherjee). A photograph of the salt formation on the surface of the upper blocks. Salt crystallization is predominantly observed in the upper block courses and within interstitial joints. This pattern is likely influenced by reduced mechanical disturbance at higher levels and by vertical microclimatic gradients linked to the absence of active ventilation. Two vertical zones can be distinguished: an upper zone, extending approximately three block courses below the ceiling, characterized by enhanced salt crystallization, and a lower zone, comprising about two block courses above the floor, where crystallization is not observable.

Greasy derivative material and discoloration phenomena can be observed in microphotographs taken by the XRF spectrometer cameras (e.g., Fig. 8, left). Evidence left by condensation droplets is clearly visible on all the lower blocks (Fig. 8, right). Furthermore, some black graffiti are also present. Salt crystallization was observed selectively in the upper block courses and on the ceilings, particularly within the interstices between blocks (Fig. 9). Efflorescence observed on the upper blocks was not sampled due to limited accessibility and the absence of sampling permission for this area.

#### In-situ XRF analysis campaign

The elements identified in all the XRF measurement are aluminum (Al), silicon (Si), phosphorus (P), sulfur (S), potassium (K), calcium (Ca), titanium (Ti), barium (Ba), manganese (Mn), and iron (Fe), as well as minor elements such as copper (Cu), zinc (Zn), strontium (Sr), zirconium (Zr), and thorium (Th). The elements detected are compatible with the composition of red Egyptian granite from the areas of Aswan published by El-Taher^50^. Aswan granites are composed mainly of alkali-feldspars (KAlSi_3_O_8_), plagioclase (NaAlSi_3_O_8_ – CaAl_2_Si_2_O_8_), quartz (SiO_2_), biotite (K(Mg, Fe)_3_(AlSi_3_O_10_)(OH)_2_), hornblende (Ca_2_(Mg, Fe, Al)_5_(Al, Si)_8_O_22_(OH)_2_). Allanite, zircon, monazite, fluorite, apatite, titanite and opaques are the main accessory minerals, while the secondary minerals are muscovite, chlorite and carbonates^5^. Granite may also contain trace amounts of zirconium (Zr), barium (Ba), and rubidium (Rb), typically found in feldspars, as well as thorium (Th) and uranium (U), which are present in accessory minerals^50–52^. XRF measurements exhibit a relatively good, matrix-dependent penetration depth; therefore, the contribution of possible patinas to the measured signal, although difficult to evaluate, is likely minimal^53^.

#### Mappings

**Fig. 10:**
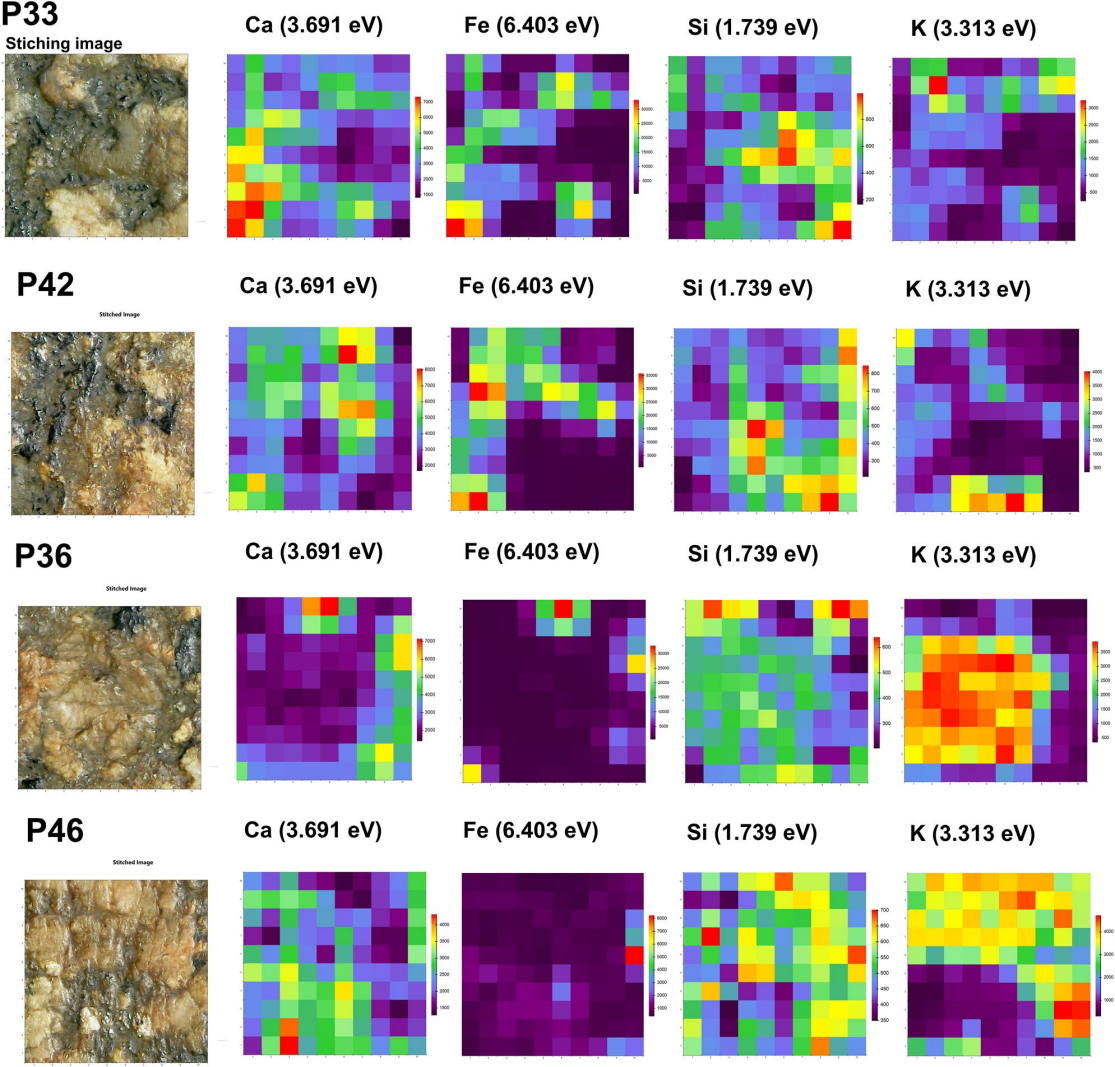
XRF mapping of measurement at points P33, P42, P36 and P46. The maps show the integrated counts of the element-specific energy lines, with signal intensity ranging from low (blue) to high (red).

Elemental distribution maps corresponding to different mineral phases are shown in Fig. 10. Further elemental maps of the same areas are presented in Supplementary Fig. S8. The maps show the integrated counts of the element-specific energy lines, with signal intensity ranging from low (blue) to high (red). Particularly noteworthy is the distribution of K: in P33 and P42, high signals correspond to the dark areas, likely representing biotite, whereas in P36 and P46, K is associated with the reddish regions, which can be attributed to alkali feldspars. This distinction is further supported by the silicon signal, which is higher in P36 and P46 and lower in P33 and P42 at the corresponding locations. A perfect correspondence of the Ca and Fe signals can be observed for the respective maps of area P36, which indicates the distribution of hornblende. While MA-XRF analysis has typically been applied to Egyptian objects for the analysis of granite in museum contexts, e.g.^40^, this study represents the first use of the technique to characterize granite minerals on-site. As demonstrated above, mappings are beneficial for discriminating phases with similar elemental composition.

#### Spot analysis

**Fig. 11:**
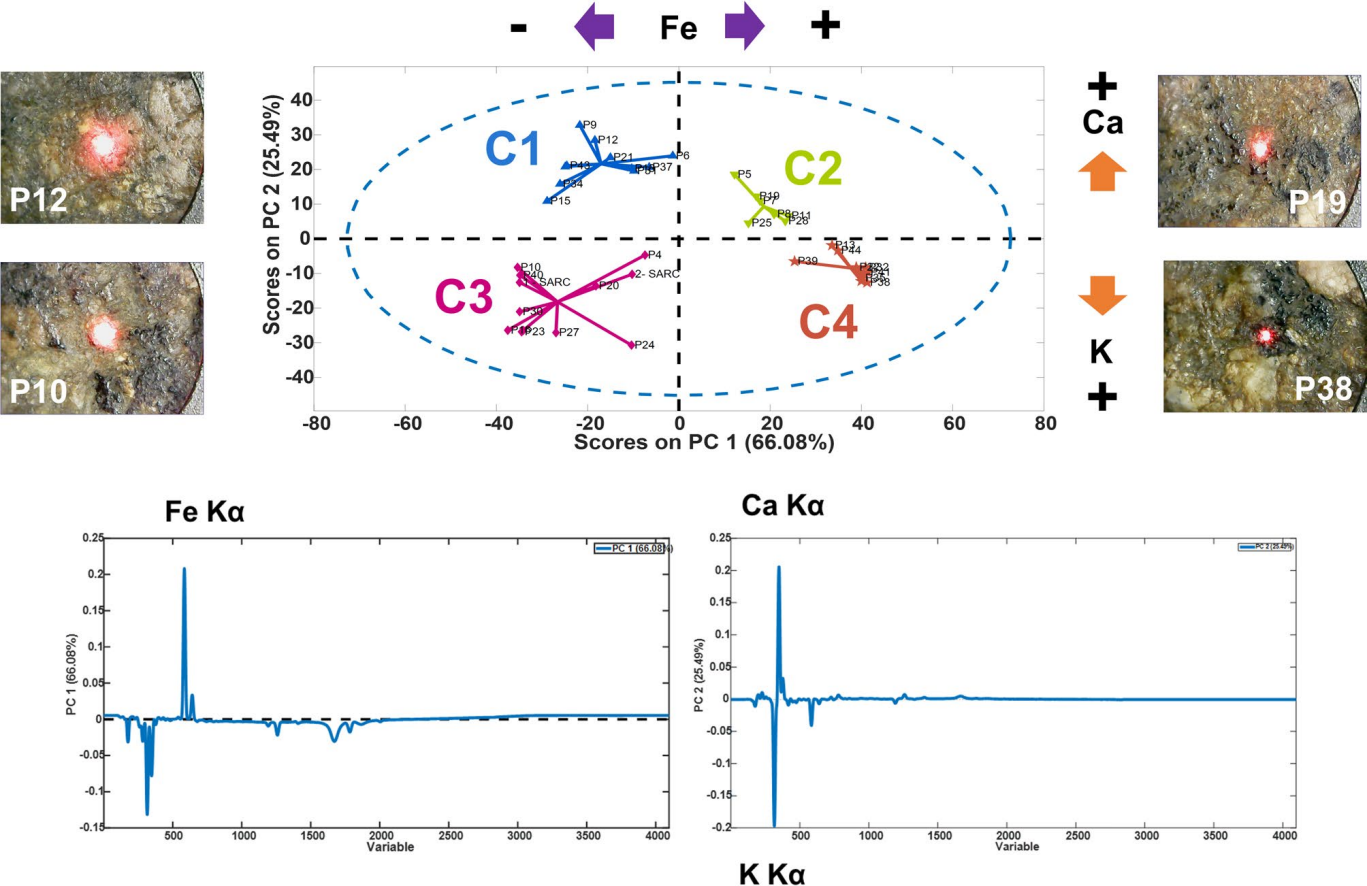
Score and loadings plots for the XRF measurements, as well as four micro-photographs representative of spectra from each data cluster. Clusters 1 and 3 indicate the white/red areas in which the signal of Fe is lower. A higher K signal in Cluster 3 indicates areas with alkali feldspar and plagioclase. C4 discriminates biotite. C2 indicates the presence of Hornblende.

For the 30 XRF spot measurements, a preliminary principal component analysis (PCA) revealed four main clusters (Fig. 11). Based on the loadings, PC1 distinguishes spectra by their iron content, with clusters C1 and C3 showing lower Fe signals (negative PC1 values) and clusters C2 and C4 showing higher Fe signals (positive PC1 values). PC2, on the other hand, considers a higher signal for Ca (positive value) and K (negative value). Fe content indicates the identification of black minerals that can correspond to biotite as well as amphibole (Hornblende). The higher K and Fe content, along with Ti and Mn, can further discriminate biotite (C4). Higher Fe and Ca signals, along with higher Si, may indicate the presence of Hornblende (C2). Clusters 1 and 3 indicate the white/red areas in which the signal of Fe is lower. A higher K signal, as in Cluster 3, indicates areas with alkali-feldspar and plagioclase.

Once the characterization of the mineral phases of the blocks has been addressed, some observations can be made regarding the deposition of salt efflorescence in the upper blocks. The crystallization phenomena may be linked to long-term weathering processes affecting granitic minerals such as feldspars and micas. This process, known as *kaolinization*, involves the hydrolysis of feldspars followed by the formation of kaolinite (Al_2_Si_2_O_5_(OH)_4_) and silica, alongside the release of alkali carbonates. As published in Schiavon et al.^54^ a CO_2_ + H_2_O-rich atmosphere, the idealized stoichiometry of the kaolinization reaction for orthoclase (K[AlSi_3_O_8_]) and albite (Na[AlSi_3_O_8_]) may be written as follows (Me = K, Na):

$$\:4Me\:\bullet\:\left[Al{Si}_{3}{O}_{8}\right]+4{H}_{2}O+2{CO}_{2}\rightleftharpoons\:{Al}_{4}{\left(OH\right)}_{8}\left[{Si}_{4}{O}_{10}\right]+2{Me}_{2}{CO}_{3}+8Si{O}_{2}.\:$$

This process is strongly influenced by pH (particularly below pH 6), feldspar surface area, and ion-exchange reactions. Although feldspar weathering is generally a slow geological process—occurring over thousands of years, the extent of the phenomenon observed on the granite surfaces of the chamber suggests that it has been accelerated under specific microclimatic conditions^55^.

Increased CO₂ concentrations and relative humidity, along with additional pollutants and contaminants, are primarily attributable to visitor presence. This activity not only increases respiration-related emissions but may also contribute to the accumulation of particulate matter, moisture, and other compounds that influence the microclimatic conditions and surface chemistry of the stone materials^56^.

After ascending a physically demanding staircase and passing through narrow corridors, visitors reach the King’s Chamber already physiologically stressed, thereby contributing significantly to the internal microclimate. Pronounced fluctuations and marked increases in CO_2_, T, and RH were recorded both in the early morning before opening—after approximately eight hours without visitors (5:30: T = 31.4 °C, RH = 75.3%, CO₂ = 858 ppm)—and during periods of active visitation (10:00: T = 32 °C, RH = 89.7%, CO_2_ = 5846 ppm). At closing time, CO_2_ concentrations exceeded the detector’s upper limit (≥ 6000 ppm) at T = 32 °C and RH = 90%.

Such elevated CO₂ levels under very high humidity conditions promote the formation of an acidic microenvironment, particularly in the presence of condensation, thereby enhancing the risk of stone deterioration. Kaolinization processes, resulting from the alteration of feldspar minerals into kaolinite, are commonly associated with mineral dissolution and the development of increased micro-porosity. The resulting modification of the pore network facilitates the migration and accumulation of dissolved salts, thereby promoting salt crystallization within the altered zones^54^. As mentioned, salt crystallization was observed predominantly in the upper block courses and in the interstices between block courses of the ceilings. This distribution may be partly attributed to continuous mechanical interaction between visitors and the lower wall surfaces, which can inhibit salt accumulation. In addition, the absence of an active ventilation system may promote vertical microclimatic gradients within the chamber, resulting in distinct temperature and relative humidity conditions. On this basis, two vertical zones can be hypothesized: an upper zone, extending approximately three block courses below the ceiling, where salt crystallization is more pronounced, and a lower zone, encompassing approximately two block courses above the floor, where such phenomena are limited (see Fig. 9). The deposition of crystals on the surfaces may be particularly favored by the variable conditions, namely lower relative humidity, which is promoted by higher temperatures in the upper regions of the chamber. These conditions enhance evaporation and lead to the deposition of crystals^55^.

As a first step, based on the literature, the conservation of the two chambers may begin with desalinating the surfaces. The methods must be tailored to the specific natural stone substrates, to the types of salts present, and to the local conditions of each area, particularly with regard to the localized stability of the stone^37,57^. Various approaches are possible, ranging from gentle mechanical removal of crystals with soft brushes^53^ to the application of tailored poultices for desalination^58^. However, while those treatments can be effective in varying degrees, understanding the cycles of dissolution and crystallization of different salt phases, together with continuous monitoring of ambient conditions, is the most effective approach to preserve the chambers. Micro-environmental control is a method to prevent or minimize damage, which can be achieved by defining RH–T ranges where phase transitions do not occur. Thus, to mitigate salt crystallization, environmental conditions in both chambers should prioritize stable, controlled temperatures (air and surface) and air relative humidity. Given the presence of two distinct climate zones in the King’s Chamber, an effective air-circulation system would help create more uniform conditions throughout space, thereby reducing the risk of condensation. Adequate air velocity supports proper circulation and helps maintain homogeneous environmental parameters; stagnant zones may promote microbial growth or allow acidic solutions to interact with the stone surfaces^55^. Conversely, excessively high air velocity should be avoided, as it may damage the artifacts by causing erosion or increasing the deposition of particulate matter on the surfaces.

As an outlook, further analytical investigations and continued environmental monitoring are planned to achieve a more comprehensive understanding of the site’s microclimatic dynamics and to support the development of targeted preventive conservation strategies aimed at safeguarding the integrity of this invaluable cultural heritage. The installation of data loggers for temperature and relative humidity has been completed, and systematic monitoring of CO_2_ concentrations will continue in order to document ongoing environmental fluctuations within the chambers. The combined evaluation of these parameters, together with in situ observations of salt formation, will provide deeper insight into crystallization–dissolution cycles and their temporal variability, thereby strengthening the scientific basis for evidence-based preventive conservation measures.

## Conclusion

The Queen’s and King’s chambers of the Khufu Pyramid present a complex interaction with the surrounding environment. The specific types of stone in both chambers exhibit different issues. Halite has been identified as the major phase responsible for the efflorescence observed in the Queen’s Chamber. No sulfation phenomena, namely gypsum-rich crusts, were identified in the black areas of the Thieves’ Corridor. In contrast, kaolinization processes affecting the granite blocks of the King’s Chamber likely promote mineral alteration and increased micro-porosity, facilitating water circulation and salt transport and thereby favouring salt precipitation in the upper blocks.

This study constitutes an important step toward a comprehensive interdisciplinary investigation of all surfaces in the Queen’s and King’s Chambers, supporting both the planning of eventual restoration–conservation interventions and the development of informed preventive conservation strategies. These aspects will be addressed in detail in subsequent publications.

Moreover, in situ non-destructive methods such as XRF have proven to be important tools for characterizing and monitoring historical surfaces and can be used to detect enrichments of Cl-based phases before efflorescence forms. Thus, in the Queen’s Chamber, a more selective sampling strategy was possible. The use of an imaging setup enabled a completely non-destructive characterization of the granite’s mineral phases in the King’s Chamber. A chemometric approach, based on PCA of XRF spot measurements, proved to be an efficient method for evaluating the dataset and identifying and characterizing the mineral components of the granite.

## Supplementary Information

Below is the link to the electronic supplementary material.

Supplementary Material 1

## Data Availability

Data will be made available on request and after permission from the Egyptian Ministry of Tourism and Antiquities.
