# Supplementary material for: Material characterization of stone surfaces in the inner chambers of the Khufu (Cheops) Pyramid: towards informed conservation strategies
Source: Sci Rep. 2026 Apr 16;16:12586. doi: 10.1038/s41598-026-48805-8 (PMC13087131; doi:10.1038/s41598-026-48805-8)
Supplement: Supplementary file 1 — Supplementary Material 1 [file 41598_2026_48805_MOESM1_ESM.docx]

**Material Characterization of Stone Surfaces in the Inner Chambers of the Khufu Pyramid: Towards Informed Conservation Strategies**

**Supplementary information**

**Supplementary Table S1. Sample descriptions.**

| **Num.** | **Description** |
| --- | --- |
| Sample 1 (S1) | Naturally detached material/**Incoherent** material from the flaking part of the ground of the queen chamber entrance. |
| Sample 2 (S2) | Naturally detached material/**Incoherent** material taken from the ground from the middle area of the thief corridor. |
| Sample 3 (S3) | Naturally detached material/Incoherent material from the flaking ceiling of the Queen's chamber. |
| Sample 4 (S4) | Solid salt sample taken in the middle area of the niche/thief corridor, from a lower block. |
| Sample 5 (S5) | Fluffy crystal salt taken in the middle area of the niche/thief corridor, from an upper block. |
| Sample 6 (S6) | Long linear salt strips taken from the upper block at the end of the thief corridor. |
| Sample 7 (S7) | Naturally detached material/Incoherent material from the entrance of the queen chamber. |
| Sample 8 (S8) | Crust taken from the upper block of the third line at the end of the thief corridor. |
| Sample 9 (S9) | A mixture of salt and sand/dust powder sample was scratched from the upper block of the western wall of the queen’s chamber |


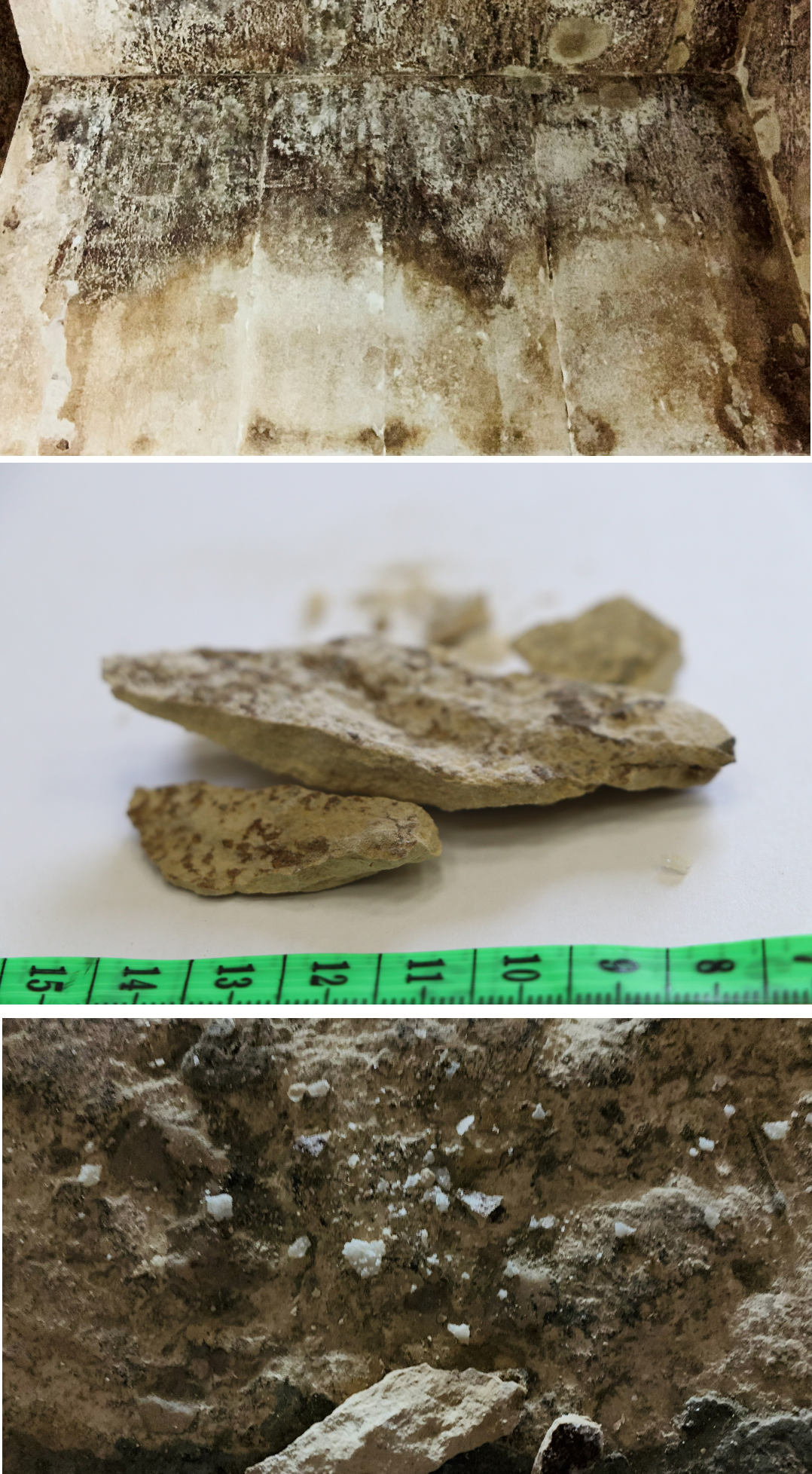


**Supplementary Figure S1.** Overview of salt formation on the ceiling of the Queen’s Chamber (upper), image of Sample S3 (middle), and salts agglomerate on the floor after detachment (lower). The material consists of naturally detached, incoherent material originating from the flaking ceiling surface.


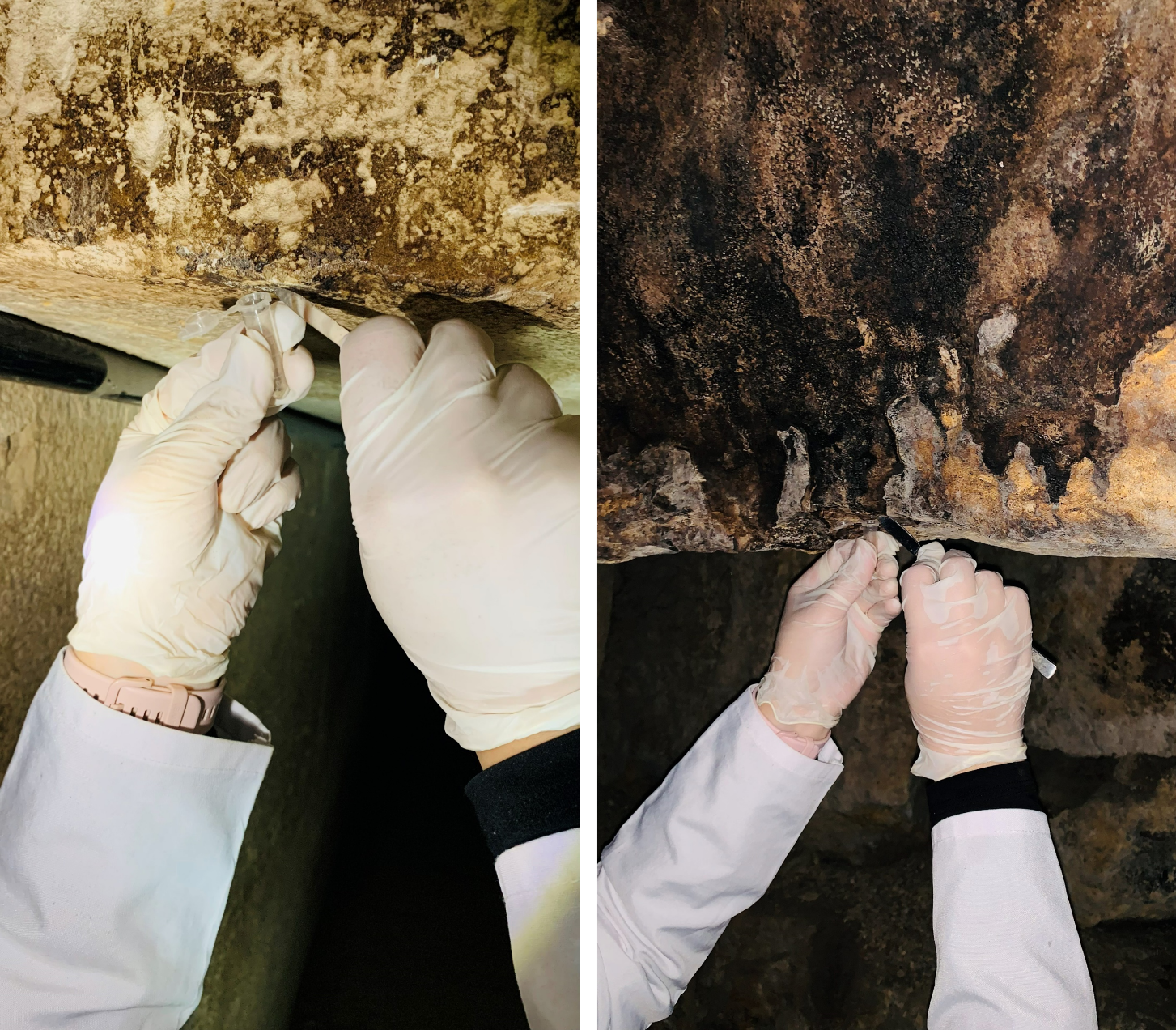


**Supplementary Figure S2.** Sampling documentation for sample S7 collected at the entrance of the Queen’s Chamber (left) and sample S8 collected at the end of the Thieves’ Corridor (right).

**
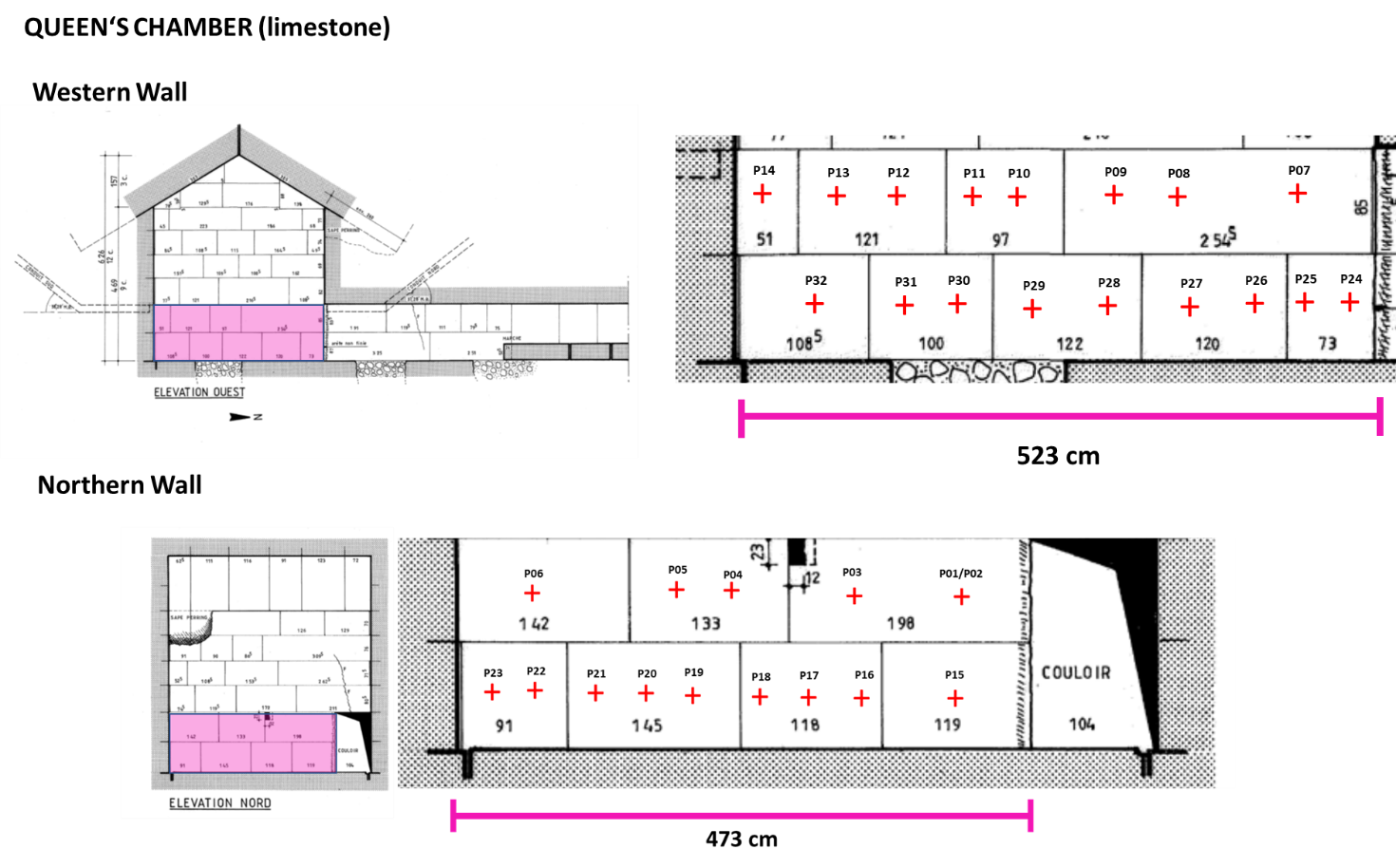
**

| **Point** | **Height (cm, from the floor)** | **Distance (cm, from the entrance/corridor)** |
| --- | --- | --- |
| **P01/02** | 129 | 63 |
| **P03** | 129 | 94 |
| **P04** | 129 | 252 |
| **P05** | 132 | 295 |
| **P06** | 132 | 341 |
| **P07** | 132 | 56 |
| **P08** | 132 | 94 |
| **P09** | 135 | 131 |
| **P10** | 135 | 169 |
| **P11** | 134 | 233 |
| **P12** | 134 | 312 |
| **P13** | 134 | 360 |
| **P14** | 134 | 405 |
| **P15** | 80 | 76 |
| **P16** | 85 | 122 |
| **P17** | 85 | 186 |
| **P18** | 80 | 211 |
| **P19** | 81 | 255 |
| **P20** | 80 | 307 |
| **P21** | 81 | 358 |
| **P22** | 81 | 393 |
| **P23** | 80 | 424 |
| **P24** | 82 | 54 |
| **P25** | 82 | 65 |
| **P26** | 82 | 115 |
| **P27** | 79 | 164 |
| **P28** | 80 | 209 |
| **P29** | 79 | 255 |
| **P30** | 79 | 326 |
| **P31** | 80 | 390 |
| **P32** | 80 | 420 |

**
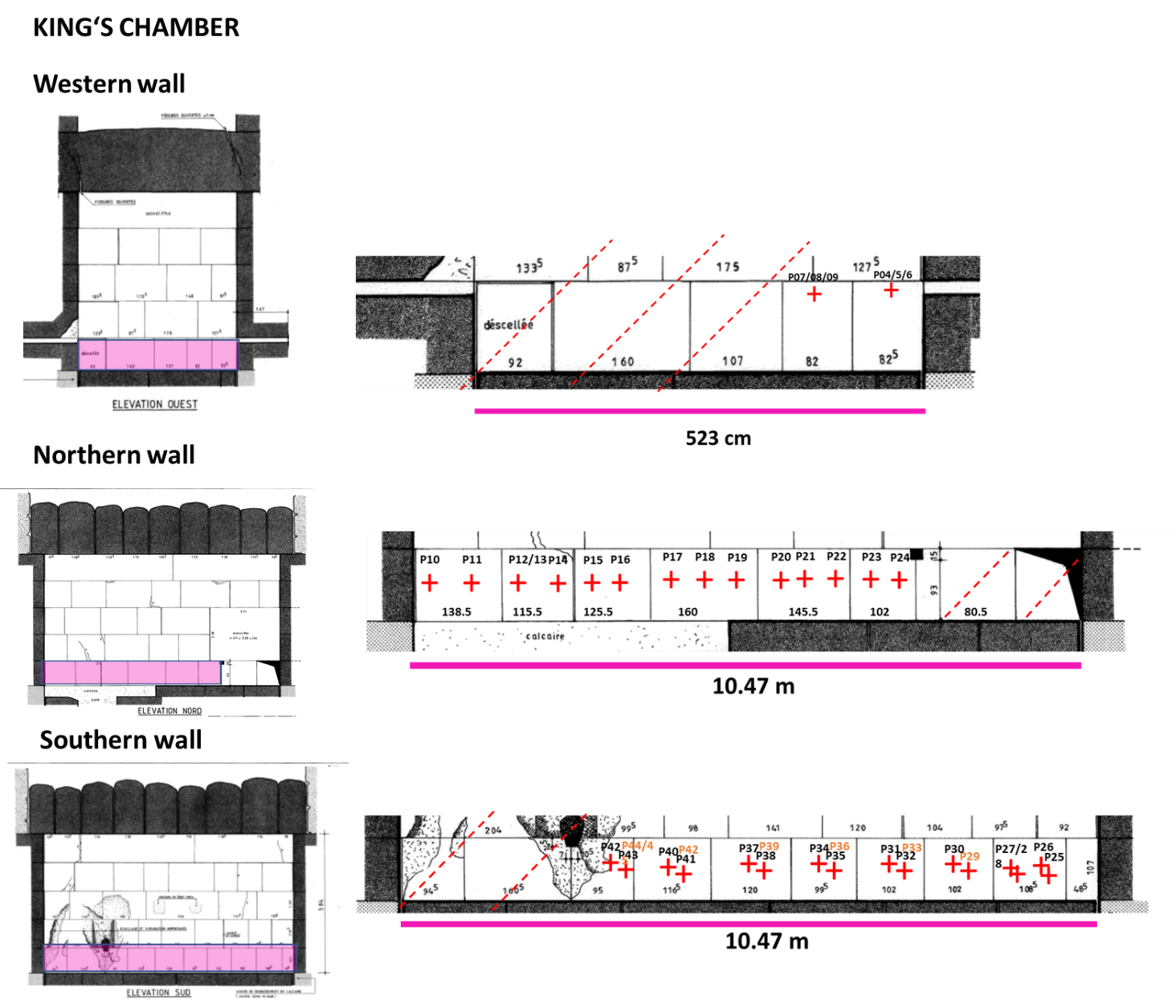
**

| **Point** | **Height (cm, from the floor)** | **Distance (cm, from the entrance/corridor)** | **Description (micro-photographs have been recorded by the XRF spectrometer for each measurement)** |
| --- | --- | --- | --- |
| **P04** | 107 | 75 | White/lighter mineral |
| **P05** | 107 | 75 | Black/darker mineral |
| **P06** | 107 | 75 | White/grey/lighter mineral |
| **P07** | 108 | 113 | White/grey/lighter mineral |
| **P08** | 108 | 113 | White/grey/lighter mineral |
| **P09** | 104 | 113 | White/grey/lighter mineral |
| **P10** | 98 | 79 | White/lighter mineral |
| **P11** | 97 | 120 | White/grey/lighter mineral |
| **P12** | 98 | 188 | Black/darker mineral |
| **P13** | 98 | 188 | Black/darker mineral |
| **P14** | 98 | 284 | White/grey/lighter mineral |
| **P15** | 98 | 313 | Black/darker mineral |
| **P16** | 97 | 353 | White/grey/lighter minerall |
| **P17** | 97 | 400 | White/grey/lighter mineral |
| **P18** | 97 | 403.5 | White/grey/lighter mineral |
| **P19** | 97 | 416.5 | Black/darker mineral |
| **P20** | 97 | 562 | White/grey/lighter mineral |
| **P21** | 97 | 620 | White/pink/lighter mineral |
| **P22** | 97 | 620.5 | Black/grey/darker mineral |
| **P23** | 97 | 698 | Black/grey/darker mineral |
| **P24** | 96 | 746 | White/grey/lighter mineral |
| **P25** | 97 | 76.5 (B1+28 cm) | White/lighter mineral |
| **P26** | 98 | 75.5 (B1+27 cm) | White/grey/lighter minera |
| **P27** | 98 | 109.5 (B1+61 cm) | White/lighter mineral |
| **P28** | 100 | 109.5 (B1+61 cm) | White/pink mineral |
| **P29** | 92 | 231 (B1+B2+74 cm) | Map (10x10 mm) |
| **P30** | 92 | 231 (B1+B2+74 cm) | White/pink mineral |
| **P31** | 92 | 289.5 (B1+B2+B3+30.5 cm) | White/pink minera |
| **P32** | 92 | 288 (B1+B2+B3+29 cm) | Black/darker mineral |
| **P33** | 92 | 288 (B1+B2+B3+29 cm) | Map (10x10 mm) |
| **P34** | 92 | 378 (B1+B2+B3+ B4+17 cm) | White/lighter mineral |
| **P35** | 92 | 379 (B1+B2+B3+ B4+18 cm) | Black/darker mineral |
| **P36** | 92 | 379 (B1+B2+B3+ B4+18 cm) | Map (10x10 mm) |
| **P37** | 92 | 484.5 (B1+B2+B3+ B4+B5+24 cm) | White/grey/lighter mineral |
| **P38** | 92 | 482.5 (B1+B2+B3+ B4+B5+22 cm) | Black/darker mineral |
| **P39** | 92 | 482.5 (B1+B2+B3+ B4+B5+22 cm) | Map (10x10 mm) |
| **P40** | 92.5 | 593 (B1+B2+B3+ B4+B5+B6+12.5 cm) | White/grey/lighter mineral |
| **P41** | 93.5 | 594 (B1+B2+B3+ B4+B5+B6+13.5 cm) | Black/darker mineral, Block 7 from the WWall |
| **P42** | 93.5 | 594 (B1+B2+B3+ B4+B5+B6+13.5 cm) | Map (10x10 mm) |
| **P43** | 93 | 702 (B1+B2+B3+ B4+B5+B6+B7+5 cm) | White/grey/lighter mineral |
| **P44** | 94 | 700 (B1+B2+B3+ B4+B5+B6+B7+3 cm) | Black/darker mineral |
| **P45** | 94 | 700 (B1+B2+B3+ B4+B5+B6+B7+3 cm) | Map (10x10 mm) |
| **P46** | 94 | 700 (B1+B2+B3+ B4+B5+B6+B7+3 cm) | Map (10x10 mm) |

**Supplementary Figure S3.** Overview of XRF measurement locations on the walls of the Queen’s and King’s Chambers. The marked points indicate areas where XRF analysis was performed, aligned with architectural elements for spatial reference and reproducibility. The measurement locations have been accurately mapped and documented to enable future analyses to be conducted in the same areas.


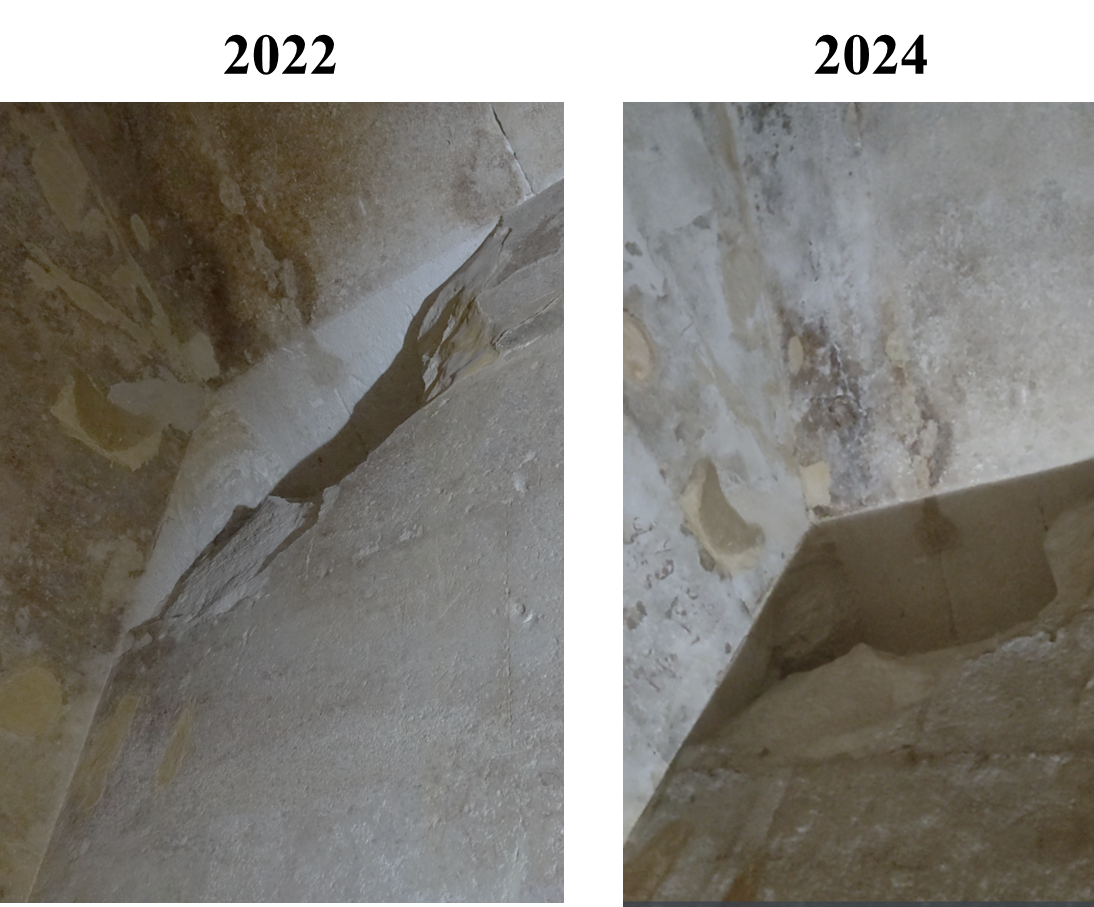


**Supplementary Figure S4.** Upper corner of the ceiling of the Queen’s Chamber in 2022 (left) and 2024 (right). An increase in salt efflorescence is clearly visible (see details indicated by the orange arrow).

**
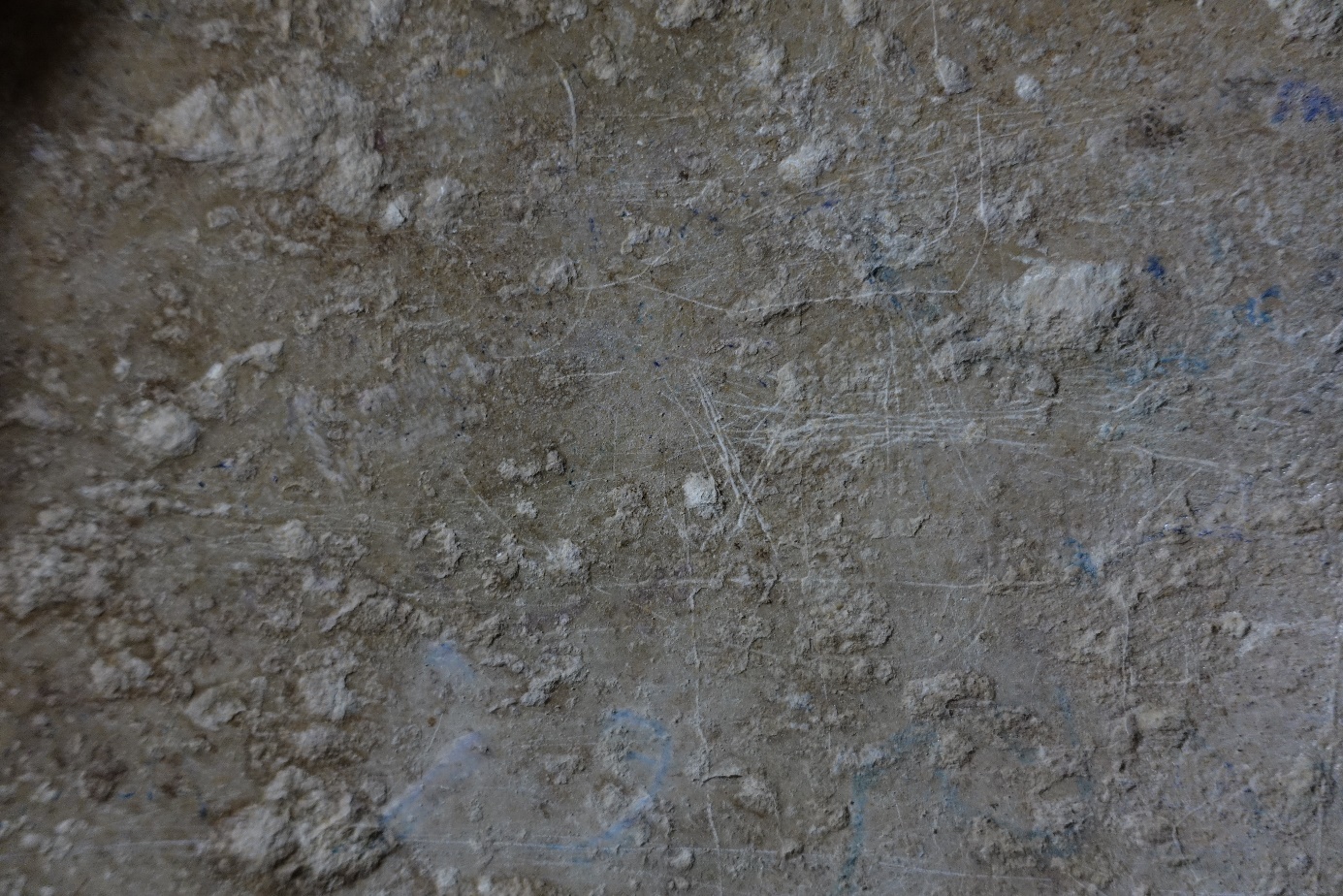
**
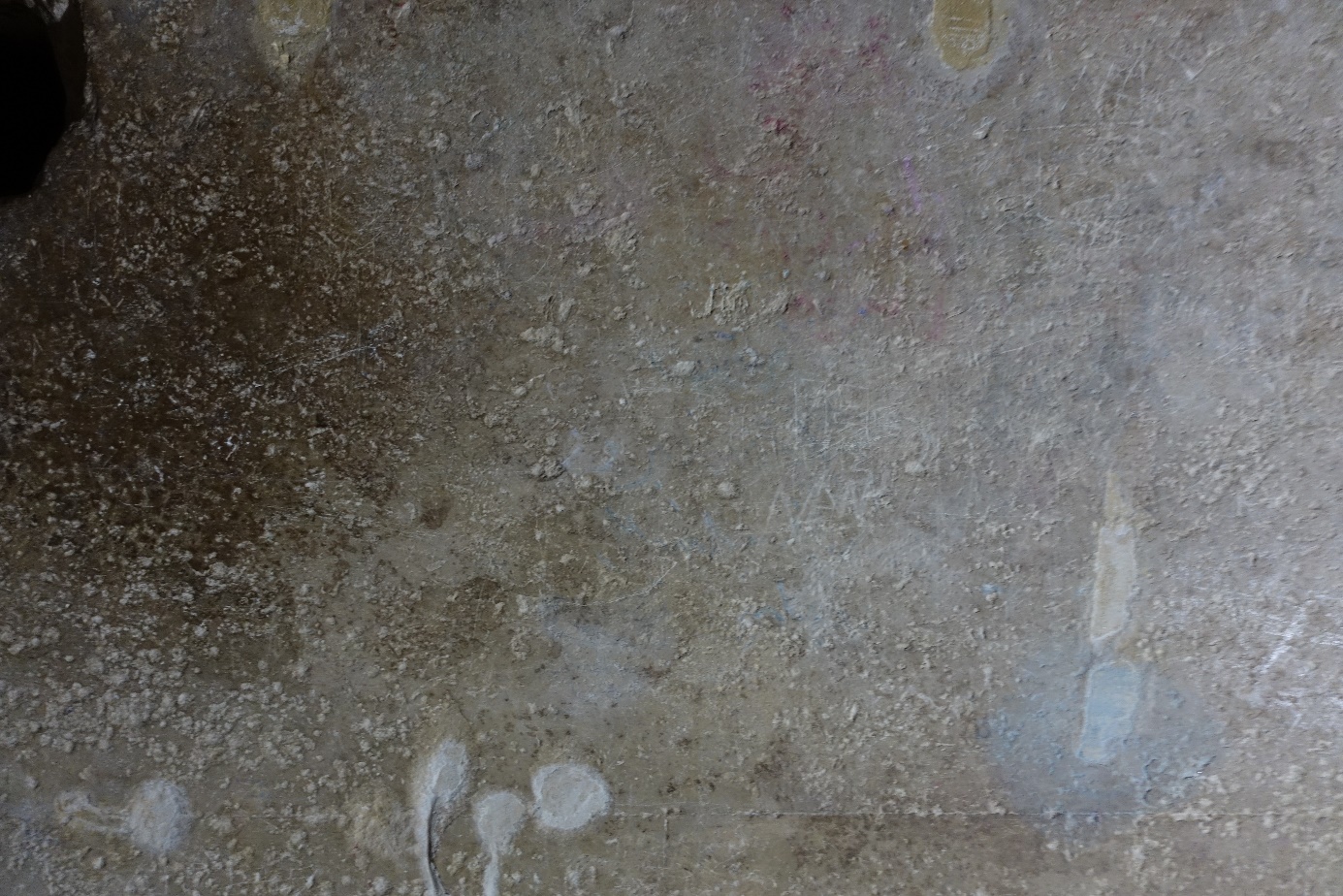


**Supplementary Figure S5. Documentation of the** anthropogenic alterations on the northern wall of the Queen’s chamber, including graffiti (blue, green, and red residues) and scratches.

**Supplementary Table S2** SEM-EDX semiquantitative elemental compositions for samples S1 to S9 (mass%).

| **Sample number and description** | **C**  **(Kα)** | **O**  **(Kα)** | **Na (Kα)** | **Mg (Kα)** | **Al (Kα)** | **Si (Kα)** | **Sr (Kα)** | **P**  **(Kα)** | **S (Kα)** | **Cl (Kα)** | **K (Kα)** | **Ca (Kα)** | **Fe (Kα)** |
| --- | --- | --- | --- | --- | --- | --- | --- | --- | --- | --- | --- | --- | --- |
| **S1** - Incoherent material – ground Queen’s chamber | 42,82 | 36,74 | 3,74 | 0,85 | - | 1,22 | 0,16 | 3,50 | 2,55 | 1,99 | 0,20 | 6,23 | - |
| **S2** - Incoherent material – ground thief corridor | 27,43 | 40,51 | 6,00 | - | 0,84 | 2,07 | - | 5,57 | 3,97 | 2,87 | 0,51 | 10,21 | - |
| **S3** - Incoherent material - ceiling Queen’s chamber | 17,77 | 53,71 | 3,77 | 1,26 | 1,58 | 2,58 | - | 3,50 | 2,61 | - | - | 13,23 | - |
| **S4** - Salt powder | 31,23 | 24,63 | 20,00 | - | - | - | - | 2,77 | 1,56 | 18,14 | - | 1,16 |  |
| **S5** - Salt powder | 0,50 | 18,22 | 47,39 | - | - | - | - | - | - | 33,40 | - | 0,50 | - |
| **S6** - Salt powder | 0,75 | 12,23 | 47,40 | - | - | - | - | - | - | 39,16 | - | 0,46 | - |
| **S7** - Brown crust | 16,47 | 46,79 | 4,98 | - | - | 2,09 | - | 11,16 | 6,19 | 3,22 | - | 9,10 | - |
| **S8** - Black crust | 39,51 | 44,9 | 1,58 | 0,47 | 0,31 | 3,03 | - | - | 0,58 | 0,53 | - | 8,75 | 0,32 |
| **S9** – Deposit of dust/salt powder | 41,06 | 40,95 | 3,95 | 0,30 | 0,49 | 5,15 | - | 0,13 | 0,12 | 1,73 | 0,18 | 30,26 | 2,04 |


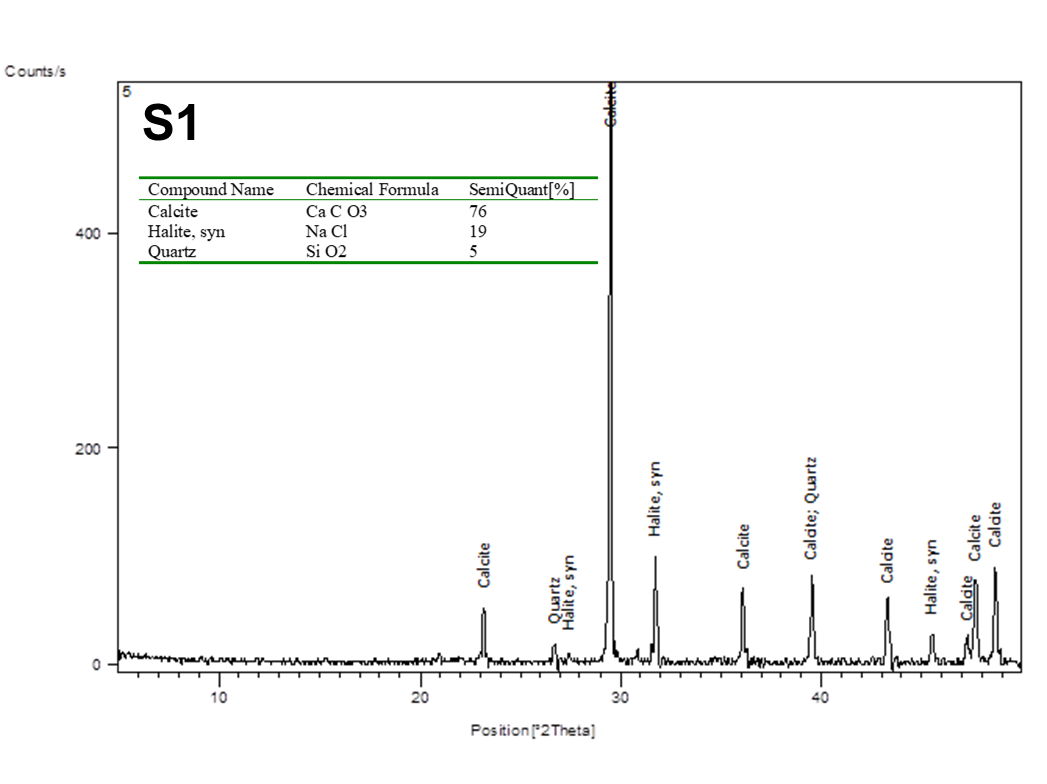

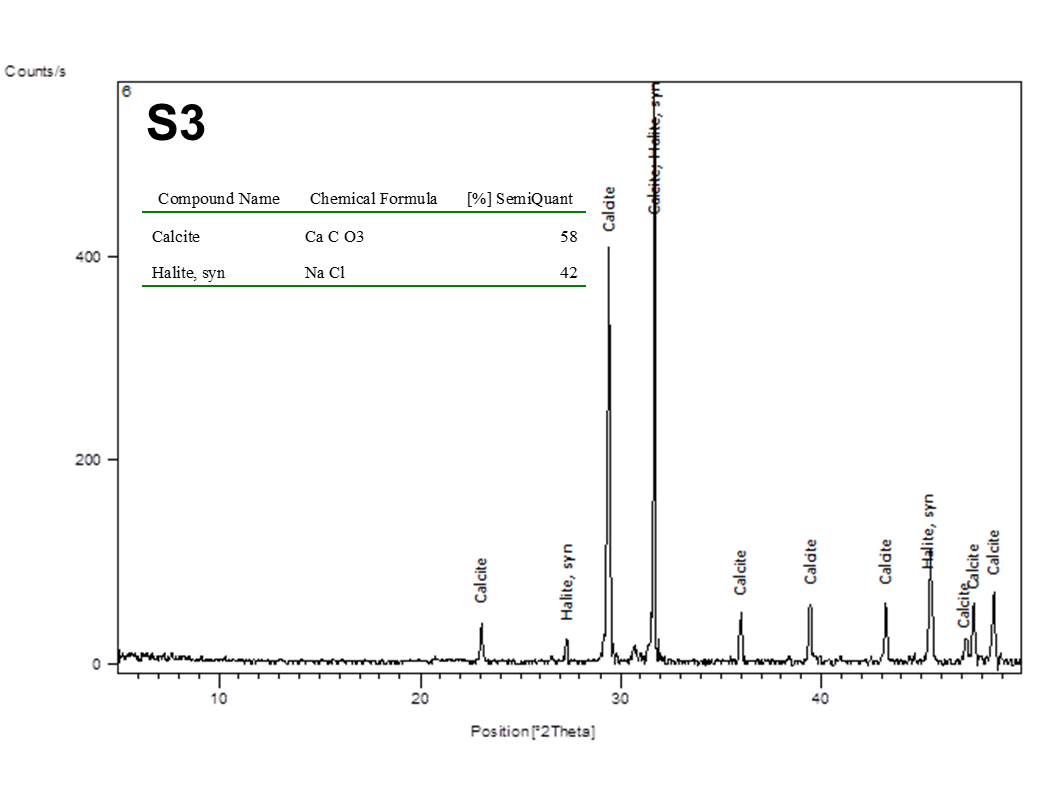

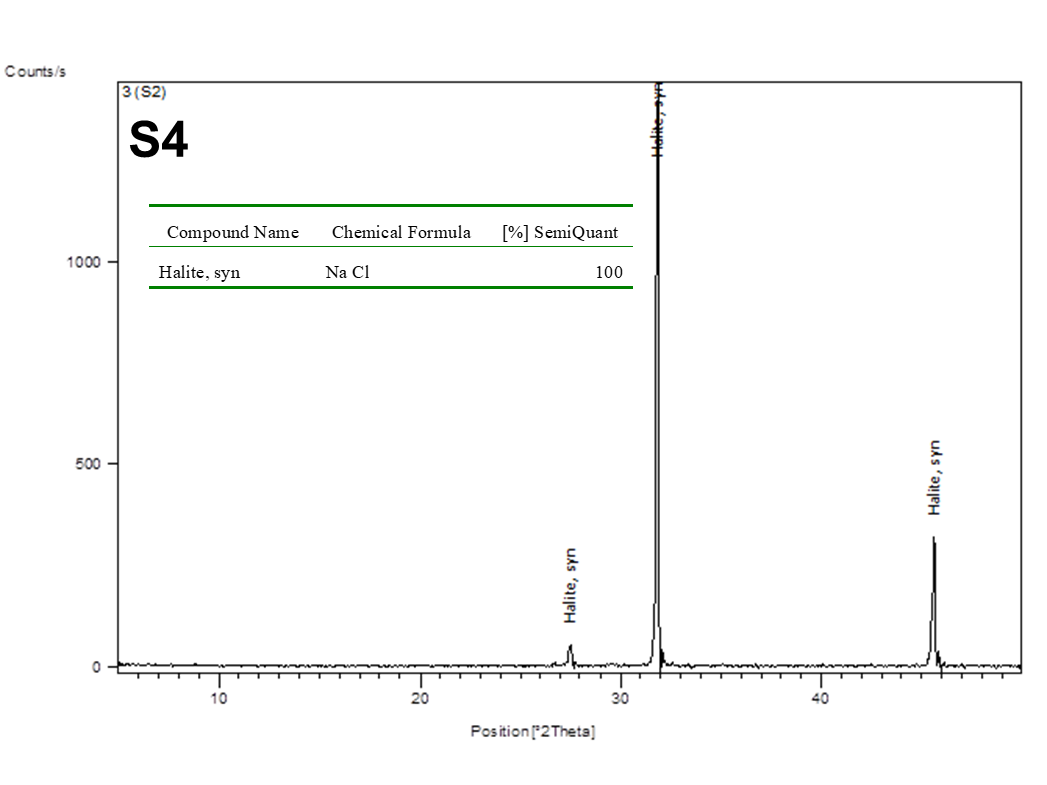

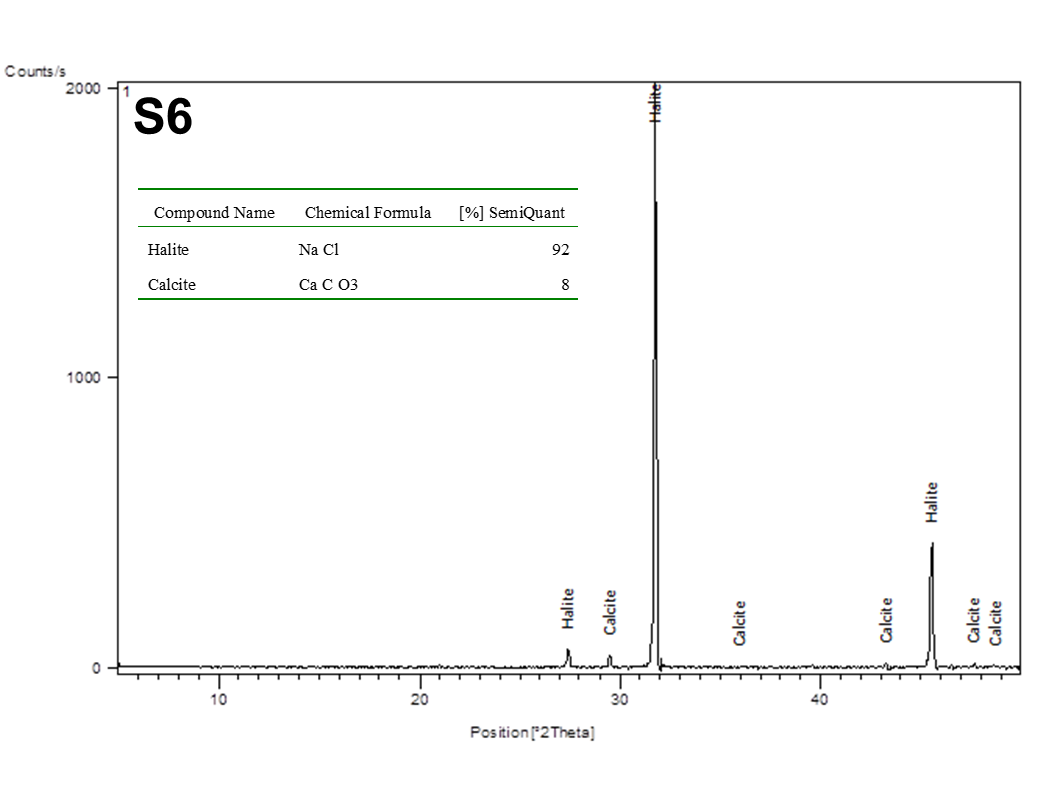


**
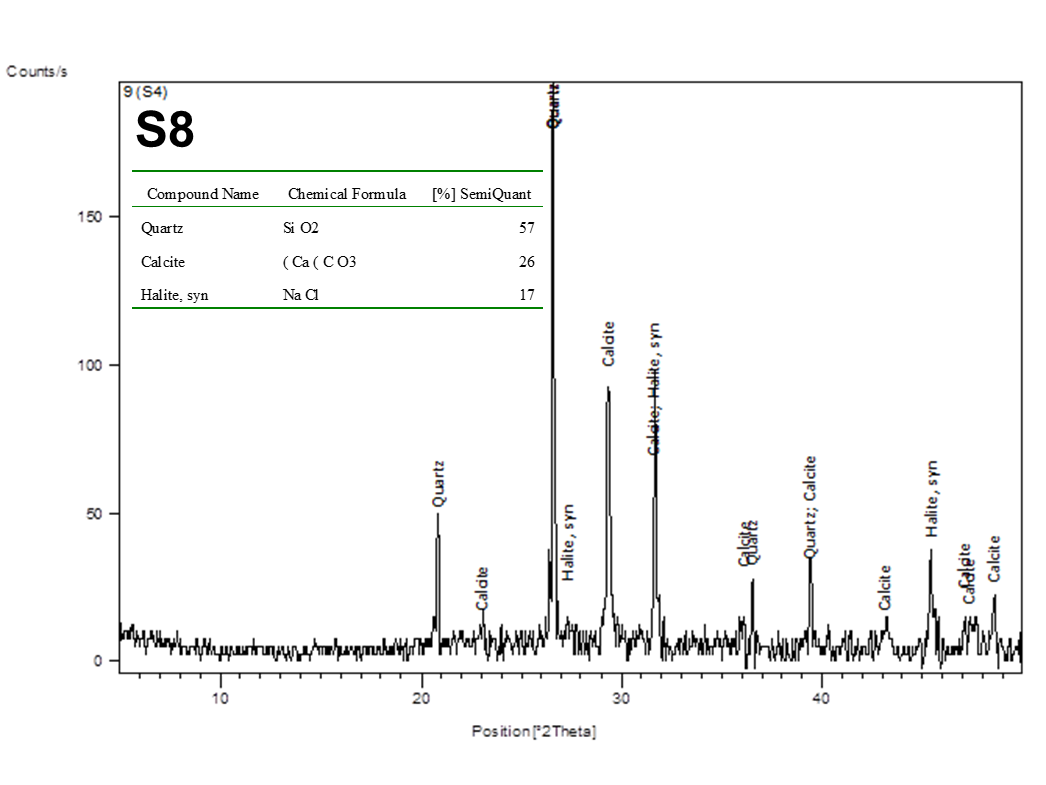
**

**Supplementary Figure S7. XRD spectra acquired for samples S1, S3, S4, S6, and S8.**

**
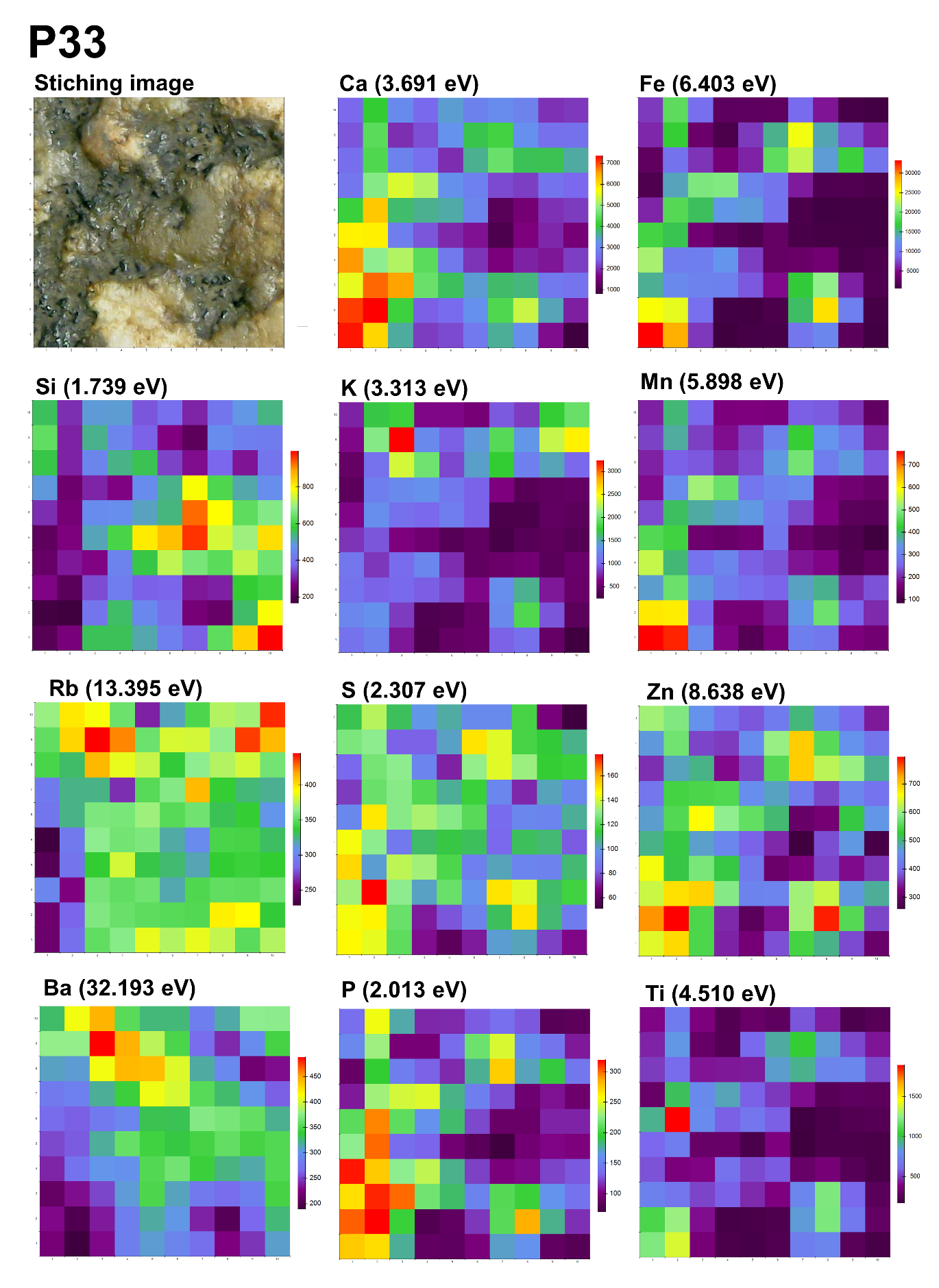
**

**
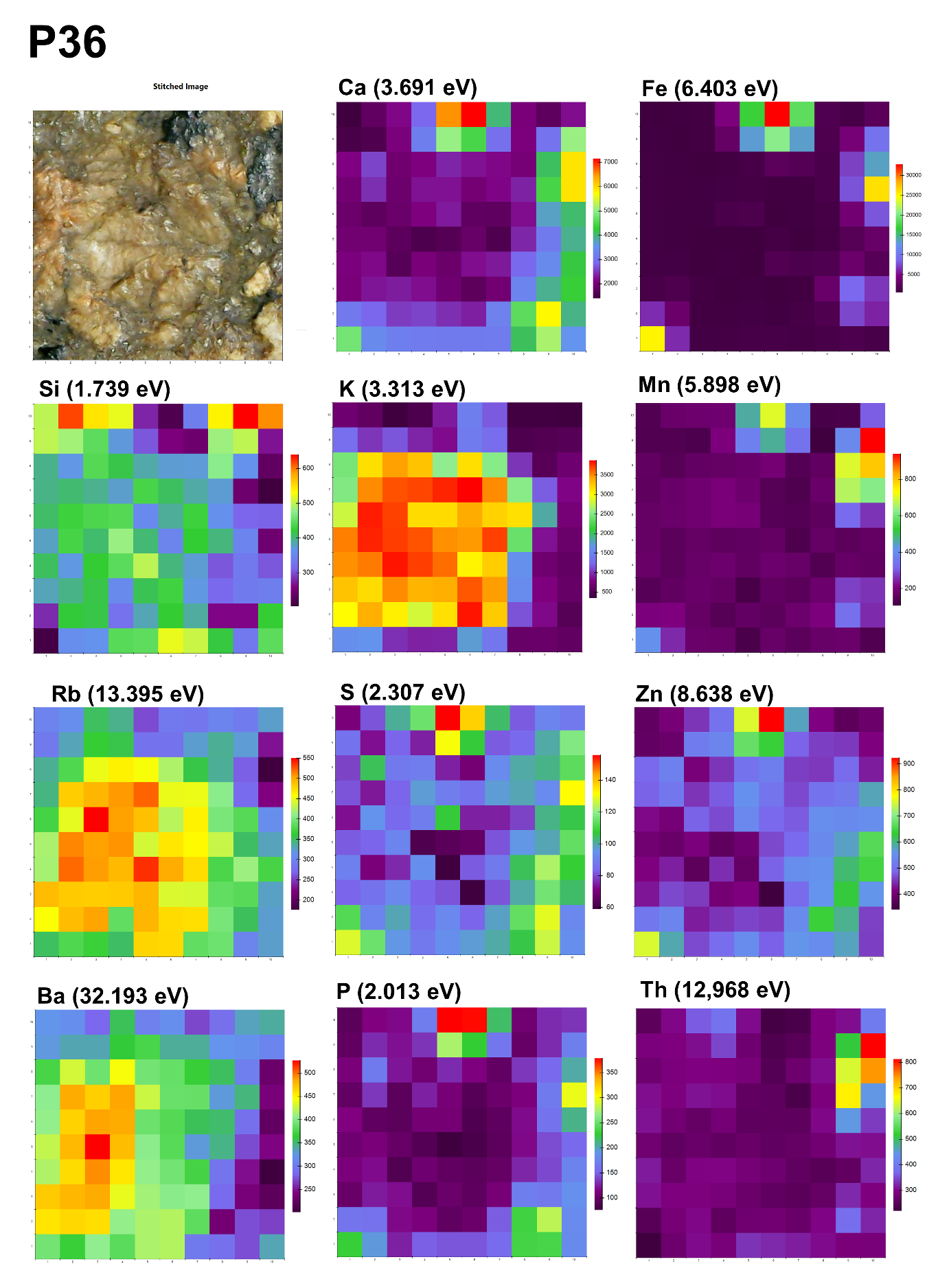
**

**
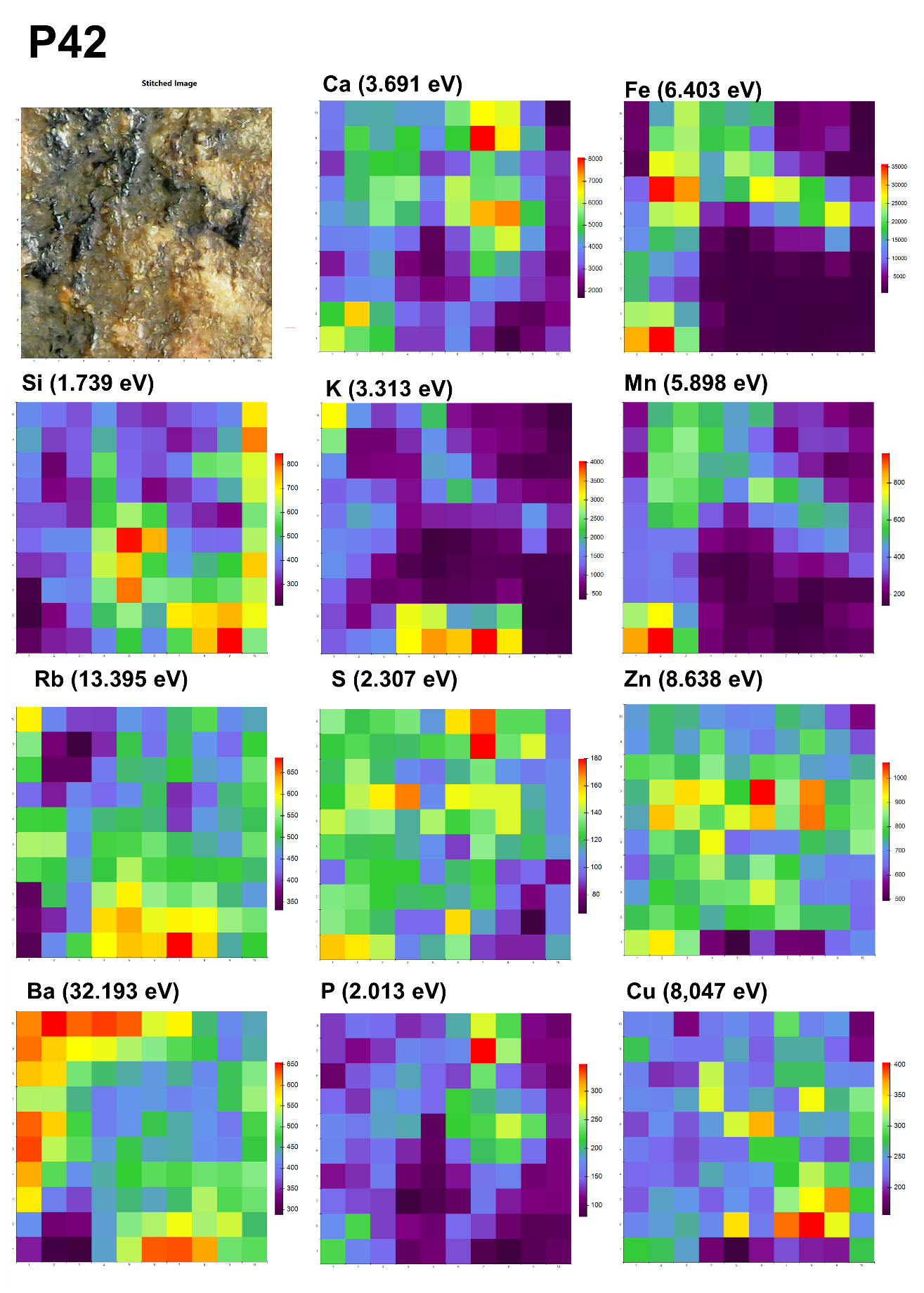
**

**
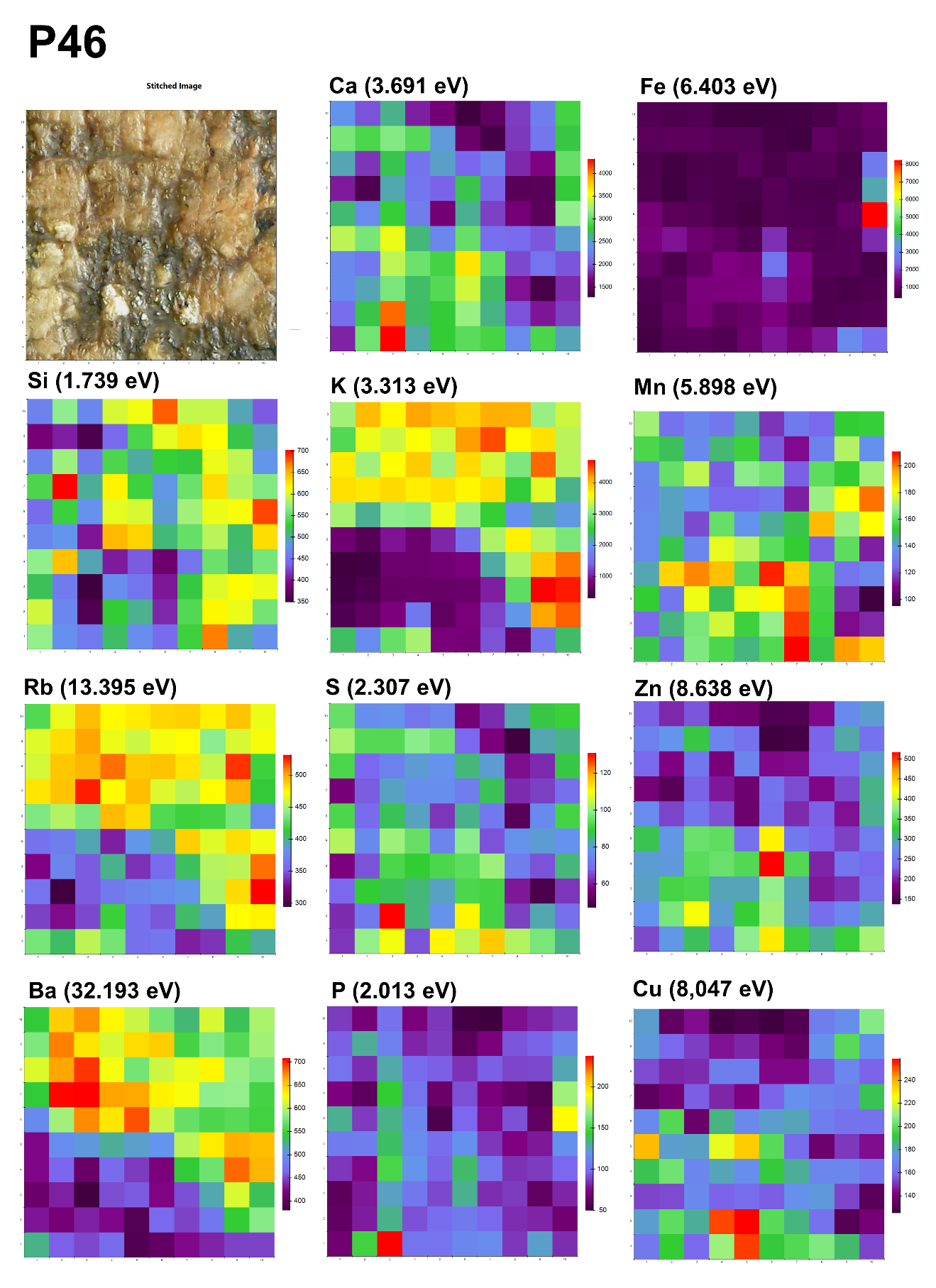
**

**Supplementary Figure S8.** XRF mapping of measurement at points P33, P42, P36 and P46. The maps show the integrated counts of the element-specific energy lines, with signal intensity ranging from low (blue) to high (red).
